# Supplementary material for: Remote home cardiotocography: A systematic review and meta-analysis
Source: PLOS Digit Health. 2026 Jan 12;5(1):e0001184. doi: 10.1371/journal.pdig.0001184 (PMC12795381; doi:10.1371/journal.pdig.0001184)
Supplement: S2 Table — (DOCX) [file pdig.0001184.s002.docx]

**S2 Table:** Search Strategy.

Database: MEDLINE and Embase

| Set | Search Term |
| --- | --- |
| S1 | Cardiotocogram OR cardiotocography OR CTG OR fetal monitoring OR maternal-fetal monitoring OR materno-fetal monitoring OR monitor* |
| S2 | obstetrics OR prenatal care OR pregnancy OR prenatal* OR antenatal* OR antepartum* OR pregnan* OR obstetric* |
| S3 | remote* OR telemetry OR telemonitoring OR telemedicine OR ambulatory OR home OR wear* OR wireless OR online OR tele* OR digital |
| S4 | fetal OR fetus |
| S5 | S1 and S2 and S3 and S4 and S4 |
