# Supplementary material for: Remote home cardiotocography: A systematic review and meta-analysis
Source: PLOS Digit Health. 2026 Jan 12;5(1):e0001184. doi: 10.1371/journal.pdig.0001184 (PMC12795381; doi:10.1371/journal.pdig.0001184)
Supplement: S3 Table — (DOCX) [file pdig.0001184.s003.docx]

**S3 Table:** Critical appraisal checklist for observational studies.

| **Domain 1: Generic quality standards** | |
| --- | --- |
| 1. Does the study address an appropriate and clearly focused question? | *Yes/no/unclear/NA* |
| 1. Was the study design appropriate to meet study aims? | *Yes/no/unclear/NA* |
| 1. Was the study size appropriate to meet study aims | *Yes/no/unclear/NA* |
| 1. Was there evidence that the authors had NOT been influenced by vested interest? | *Yes/no/unclear/NA* |
| 1. Confirmed ethical approval? | *Yes/no/unclear/NA* |
| 1. Did they take informed consent from all parties involved? Or appropriate waiver stated? | *Yes/no/unclear/NA* |
| 1. Were the methods described of sufficient detail to enable study replication? | *Yes/no/unclear/NA* |
| 1. Have they reported on all the outcomes stated within aims/methods? (Is there a risk of selective reporting) | *Yes/no/unclear/NA* |
| 1. Did the authors report on study limitations? | *Yes/no/unclear/NA* |
| 1. Did the author report on generalisability/external validity? | *Yes/no/unclear/NA* |
| **Domain 2: Patient/Participant selection:  Risk of bias** | |
| 1. If simulated patients were used, were they as representative? | *Yes/no/unclear/NA* |
| 1. Was the level of expertise of reviewers and operators adequately described? | *Yes/no/unclear/NA* |
| 1. Was an appropriate method of participant selection used? | *Yes/no/unclear/NA* |
| 1. Did the study avoid inappropriate exclusions of either participants or patients? | *Yes/no/unclear/NA* |
| 1. Could the selection of participants have introduced bias? | *RISK: High, low, unclear* |
| **Domain 3: Index test(s) (if more than 1 index test was used, please complete for each test:   Risk of bias** | |
| 1. Were the reviewers blinded to the health state of the patient? OR were they blinded to the mode of the transmission used? | *Yes/no/unclear/NA* |
| 1. Were the outcome measures appropriate to the study aims? | *Yes/no/unclear/NA* |
| 1. If subjective outcomes were reported, was an appropriate score or scale used e.g. Likert scores? | *Yes/no/unclear/NA* |
| 1. Were appropriate statistical tests used? | *Yes/no/unclear/NA* |
| 1. Were statistics reported with confidence intervals? | *Yes/no/unclear/NA* |
| 1. Could the conduct or interpretation of the index test have introduced bias? | *RISK: High, low, unclear* |
| **Domain 4: Reference standard: Risk of bias** | |
| 1. Was a reference standard used? | *Yes/no/unclear/NA* |
| 1. Is the reference standard likely to correctly classify the target condition? 2. Were the reference standard results interpreted without knowledge of the results of the index test? (Were the participants/reviewers blinded to the comparison group outcomes) | *Yes/no/unclear/NA*  *Yes/no/unclear/NA* |
| 1. Could the reference standard, its conduct, or its interpretation have introduced bias? | *RISK: High, low, unclear/ NA* |
| **Domain 5: Flow and timing: Risk of bias** | |
| 1. Was there an appropriate interval between index test(s) and reference standard? | *Yes/no/unclear/NA* |
| 1. Did all patients receive a reference standard? | *Yes/no/unclear/NA* |
| 1. Did patients receive the same reference standard? | *Yes/no/unclear/NA* |
| 1. Were all patients/participants included in the analysis | *Yes/no/unclear/NA* |
| 1. Could the patient/participant flow have introduced bias? | *RISK: High, low, unclear* |
| **Domain 6: Telemedicine/ Feasibility specific concerns** | *Yes/no/unclear/NA* |
| 1. Were the sending and/or receiving environments representative of clinical practice and/or the study aims? | *Yes/no/unclear/NA* |
| 1. Was the level of cost described? | *Yes/no/unclear/NA* |
| 1. Were technical barriers to implementation described? | *Yes/no/unclear/NA* |
| **Domain 7: Concerns regarding applicability** | |
| 1. Is there concern that the reference or comparison standard used does not match the review question? | *CONCERN: Low, high, unclear, NA* |
| 1. Is there concern that the included patients do not match the review question? | *CONCERN: Low, high, unclear, NA* |
| 1. Is there concern that the mode of ultrasound, its conduct, or interpretation differ from the review question? | *CONCERN: Low, high, unclear, NA* |

Adapted from the QUADAS-2 criteria and informed by prior publication by Marsh-Feiley et al. (4,10).
