# Supplementary material for: Remote home cardiotocography: A systematic review and meta-analysis
Source: PLOS Digit Health. 2026 Jan 12;5(1):e0001184. doi: 10.1371/journal.pdig.0001184 (PMC12795381; doi:10.1371/journal.pdig.0001184)
Supplement: S4 Table — (DOCX) [file pdig.0001184.s004.docx]

**S4 Table:** Excluded studies and associated reason for exclusion.

| Author | Year | Title | Reason for the exclusion |
| --- | --- | --- | --- |
| Abburi | 2024 | Adopting artificial intelligence algorithms for remote fetal heart rate monitoring and classification using wearable fetal phonocardiography | Not home fetal monitoring |
| Ahmad | 2018 | Fetal heart rate monitoring device using condenser microphone sensor: Validation and comparison to standard devices | Not home fetal monitoring |
| Ahmed | 2024 | Emerging Paradigms in Fetal Heart Rate Monitoring: Evaluating the Efficacy and Application of Innovative Textile-Based Wearables | Wrong study design |
| Albert | 1983 | Non-invasive fetal monitoring using radiotelemetry | Not home fetal monitoring |
| Andrade | 2015 | An Integrated Sensing Platform for Remote Fetus Continuous Monitoring | Wrong publication type |
| Anonymous | 1986 | Monitoring high risk pregnancies at home | Wrong publication type |
| Anonymous | 1990 | [A method of evaluating self-monitoring at home during pregnancy] | Not full text available |
| Anonymous | 1986 | Monitoring high risk pregnancies at homeA1 - Anonymous | No full text available |
| Anonymous | 1990 | A method of evaluating self-monitoring at home during pregnancyA1 - Anonymous. | Unable to translate |
| Aquino | 2022 | Patients' and Providers' Perspectives on and Needs of Telemonitoring to Support Clinical Management and Self-care of People at High Risk for Preeclampsia: Qualitative Study. | Does not use cardiotocography |
| Axelrod | 2023 | Putting the "f"etus back in maternal-fetal telemedicine: a prospective pilot study | Wrong publication type |
| Blondel | 1996 | Fetal heart rate monitoring by telephone during pregnancy - Opinion of users and non-users of a new technology | Does not use cardiotocography |
| Bluecher | 1978 | Tocography of premature contractions by telephone | Unable to translate |
| Boehm | 1979 | Xerox telecopier transmission of fetal monitor tracings: A 4-year experience | Not home fetal monitoring |
| Boehm | 1973 | Xerox 400 telecopier and fetal monitor | Not home fetal monitoring |
| Brennan | 1996 | A system for the delivery of remote obstetrical care by telepresence | No full text available |
| Brisinda | 2015 | Magnetocardiography provides beat-to-beat monitoring of fetal rhythm in unshielded clinical ambulatory setting at the 28th week of gestation | Wrong publication type |
| Brisinda | 2005 | Multichannel mapping of fetal magnetocardiogram in an unshielded hospital setting. | Does not use cardiotocography |
| Bureev | 2016 | A mobile full-time daily dystem for fetal monitoring | Not home fetal monitoring |
| ButlerTobah | 2019 | Randomized comparison of a reduced-visit prenatal care model enhanced with remote monitoring | Does not use cardiotocography |
| Chaturvedi | 2020 | Consistent observing of fetal pulse and uterus withdrawls | No full text avialable |
| Chen | 2009 | A comparative study of a new cardiotocography analysis program | Intrapartum cardiotocography |
| Crepin | 1981 | [Telemetric transmission of fetal cardiac rhythm in monitoring high risk pregnancies (author's transl)] | Unable to translate |
| Cuneo | 2023 | Prospective Evaluation of Anti-SSA/Ro Pregnancies Supports the Utility of High Titer Antibodies and Fetal Home Monitoring for the Detection of Fetal Atrioventricular Block | Does not use cardiotocography |
| Cuneo | 2019 | Home Monitoring for Fetal Heart Rhythm During Anti-Ro Pregnancies | Does not use cardiotocography |
| Dahlberg | 1988 | A perinatal center based antepartum homecare program | No full text available |
| Dahlberg | 1995 | High-tech, high-touch perinatal home care | Wrong study design |
| Dalton | 1983 | Long distance telemetry of fetal heart rate from patients' homes using public telephone network | Wrong study design |
| Dalton | 1985 | Fetal home telemetry in Cambridge | Wrong publication type |
| Dangel | 2024 | Urgent Follow-up after Outpatient Nonstress Tests and the Potential for Fetal Monitoring at Home | Not home fetal monitoring |
| DiLieto | 2011 | The new wireless TOCOMAT system of conventional and computerized telecardiotocography | Unable to translate |
| DiLieto | 2002 | Prenatal telemedicine: clinical experience with conventional andcomputerised antepartum telecardiotocography | Not home fetal monitoring |
| DiRenzo | 1994 | The role of telemetry in perinatal monitoring | Wrong publication type |
| Ditch | 2024 | 285 Safety and performance of a cloud-based fetal and maternal monitoring system | Wrong publication type |
| Duminy | 2005 | Telemetric antenatal fetal monitoring | Wrong publication type |
| Fink | 2017 | Home- and community-based growth monitoring to reduce early life growth faltering: an open-label, cluster-randomized controlled trial. | Wrong patient population |
| Funk | 1992 | Telemetrische Kardiotokographie - Klinische Wertigkeit und Grenzen derMethode in der Routineüberwachung | Unable to translate |
| Giacoia | 1987 | Phone transmission of fetal heart rate (FHR) tracings in a rural setting | Not home fetal monitoring |
| Gnirs | 1991 | Practical telemetry and telephonic data transmission of the cadiotocogram in the obstetric clinic | Wrong study design |
| Gyselaers | 2019 | Mobile health applications for prenatal assessment and monitoring | Wrong publication type |
| Hamm | 2022 | Experience with home-based, remote non-stress tests, including automatic decision support for interpretation of reactivity | Wrong publication type |
| Hara | 1991 | Centralized fetal monitoring with telecommunication | Wrong study design |
| Hara | 1989 | The development of a computer-aided perinatal information system | Wrong study design |
| Harkey | 2014 | Assessing the clinical use of a novel, mobile fetal monitoring device | Wrong publication type |
| Heaman | 1998 | Antepartum home care for high-risk pregnant women | Wrong publication type |
| Heazell | 2023 | Antenatal fetal heart rate monitoring at home-Experience from a Tertiary Maternity Service | Wrong publication type |
| Heinrich | 1986 | Telemetric fetal monitoring using monitor BMT 914-1 | Np full text available |
| Hinton | 2024 | Quality framework for remote antenatal care: qualitative study with women, healthcare professionals and system-level stakeholders | Not home fetal monitoring |
| Hod | 2003 | Daily self-monitoring fetal assessment and transmission from home in fetal growth restriction pregnancies | Wrong study design |
| Houzedel'Aulnoit | 2018 | Development of a Smart Mobile Data Module for Fetal Monitoring in E-Healthcare | Not home fetal monitoring |
| Huddleston | 1977 | The prediction of fetal oxygenation by an on-line computer analysis of fetal monitor output | No full text available |
| Huret | 2014 | Home care of premature rupture of membranes prior to 37 weeks' gestation | Does not use cardiotocography |
| IEEE | 2014 | Development and validation of an AI-enabled mHealth technology for in-home pregnancy management | Wrong study design |
| IEEE | 2014 | An mHealth Approach to Remote Fetal Monitoring | Not home fetal monitoring |
| IEEE | 2009 | Remote Home-Based Ante and Post Natal Care | Not home fetal monitoring |
| IEEE | 2016 | 19.2 cm Flexible Fetal Heart Rate Sensor for Improved Quality of Pregnancy Life | Wrong study design |
| IEEE | 2014 | Fetal Heart Rate Monitoring System with Mobile Internet | Does not use cardiotocography |
| Ippolito | 2003 | A cost study of prenatal telemedicine | Not home fetal monitoring |
| James | 1988 | Fetal heart-rate monitoring by telephone. 2. Clinical experience in 4 centers with a commercially produced system | Wrong study design |
| Jepsen | 2025 | ‘Being at home instead of going to the hospital is Great’ – how partners experience telemonitoring of the fetal heart rate during pregnancy – A qualitative interview study. | Does not use cardiotocography |
| Kapaya | 2018 | Women's experience of wearing a portable fetal-electrocardiogram device to monitor small-for-gestational age fetus in their home environment | Does not use cardiotocography |
| Karmakar | 2025 | Consumer insights from a feasibility study on remote and extended use of a novel non-invasive wearable fetal electrocardiogram monitor | Does not use cardiotocography |
| Kelly | 1980 | Ambulant fetal monitoring | Not home fetal monitoring |
| Klapholz | 1977 | Evaluation of model 78100A adult telemetry unit for the use in fetal heart-rate monitoring | Not home fetal monitoring |
| Klein | 1994 | Long-distance foetal monitoring | Unable to translate |
| Klein | 1994 | [Fetal telemonitoring] | Unable to translate |
| Kobayashi | 2025 | Virtual telehealth visits for prenatal checkups during the COVID- 19 pandemic in Japan: a nationwide survey and feasibility study | Does not use cardiotocography |
| Kosa | 2008 | Experiences with fetal phonocardiographic telemonitoring and future possibilities | Does not use cardiotocography |
| Kovacs | 2010 | A new, phonocardiography-based telemetric fetal home monitoring system | Wrong study design |
| Kovács | 2010 | Fetal phonocardiography--past and future possibilities | Wrong study design |
| Kuhnert | 2007 | Twenty-four-hour CTG monitoring: Comparison of normal pregnancies of 25-30 weeks of gestation versus 36-42 weeks of gestation | Not home fetal monitoring |
| Kuhnert | 2001 | 24 Hour-CTG monitoring: Comparison of normal pregnancies and pregnancies with placenta insufficiency | Not home fetal monitoring |
| Kuleva | 2012 | The value of daily fetal heart rate home monitoring in addition to serial ultrasound examinations in pregnancies complicated by fetal gastroschisis | Does not use cardiotocography |
| Lempersz | 2021 | User evaluation of real-time CTG home monitoring: A pilot study | Wrong study design |
| Likitalo | 2025 | Integrating Remote Monitoring Into the Pregnancy Care: Perspectives of Pregnant Women and Healthcare Professionals | Not home fetal monitoring |
| Lillitos | 2023 | Performance of a single-ventricle home-monitoring programme: survival and predictors of adverse outcome | Does not use cardiotocography |
| Lindsay | 1990 | Patient-recorded domiciliary fetal monitoring | No full text available |
| Lowery | 2023 | OB HUB: Remote Electronic Fetal Monitoring Surveillance | Intrapartum cardiotocography |
| Lu | 2018 | Computerised Interpretation Systems for Cardiotocography for both Home and Hospital Uses | Intrapartum cardiotocography |
| Lukyanov | 2023 | EE412 Analyzing the Financial Impact of a Remote Fetal Monitoring Solution Introduction for High-Risk Pregnancies in a US Obstetrics Clinic | Wrong publication type |
| Lukyanov | 2023 | Analyzing the Financial Impact of a Remote Fetal Monitoring Solution Introduction for High-Risk Pregnancies in a US Obstetrics Clinic | Wrong publication type |
| Marien | 2022 | Assessing the feasibility of long term home electrophysiological fetal heart rate monitoring | Wrong study design |
| Marques | 2021 | IoT-Based Smart Health System for Ambulatory Maternal and Fetal Monitoring | Not home fetal monitoring |
| McCabe | 2021 | Remote monitoring of pregnant and postpartum women with COVID-19 | Wrong publication type |
| Mhajna | 2020 | Wireless, remote solution for home fetal and maternal heart rate monitoring | Not home fetal monitoring |
| Mittra | 2008 | Development of a web-based fetal monitoring system: An innovative approach | Not home fetal monitoring |
| Monincx | 2001 | Maternal health, antenatal and at 8 weeks after delivery, in home versus in-hospital fetal monitoring in high-risk pregnancies | Wrong outcomes |
| Mugyenyi | 20017 | Functionality and acceptability of a wireless fetal heart rate monitoring device in term pregnant women in rural Southwestern Uganda | Not home fetal monitoring |
| Murakami | 1992 | A new system of fetal home monitoring – Homic network (fetus) | No full text available |
| Mwakawanga | 2024 | Barriers and facilitators of fetal heart monitoring with a mobile cardiotocograph (iCTG) device in underserved settings: An exploratory qualitative study from Tanzania | Does not use cardiotocography |
| Nair | 2023 | Evaluation of the fetal heart rate monitoring with the non-invasive electrocardiography signals | Wrong publication type |
| Nakajima | 2004 | Fetal heart rate and uterine contraction during automobile driving | Not home fetal monitoring |
| Neppelenbroek | 2024 | Antenatal cardiotocography in dutch primary midwife-led care: Maternal and perinatal outcomes and serious adverse events. A prospective observational cohort study | Wrong study design |
| Nitulescu | 2015 | Integrated Wireless Sensor Network for Monitoring Pregnant Women | Not home fetal monitoring |
| Olesiak-Andryszczak | 2024 | Home ctg remote care in high-risk pregnancy | Wrong study design |
| Pan | 2000 | Clinical experience of long-distance electronic fetal heart rate monitoring system by telephone | Unable to translate |
| Parer | 1979 | Clinical experience with telemetered heart rate monitoring | Intrapartum CTG |
| Peahl | 2020 | Prenatal care redesign: creating flexible maternity care models through virtual care | Wrong publication type |
| Pilarczyk | 2020 | Diagnostic equivalency of mobile CTG devices and remote analysis to conventional on-site nonstress test | Not home fetal monitoring |
| Pitts | 2021 | Fetal Heart Rate Decelerations in Women with Sleep-Disordered Breathing | Does not use cardiotocography |
| Polvani | 1972 | [Fetal monitoring by means of radiotelemetry] | Unable to translate |
| Quemere | 2000 | Obstetrical home monitoring: The Nancy experience from 1992-1997 | Unable to translate |
| Reece | 1992 | A controlled trial of self nonstress test versus assisted nonstress test I the evaluation of fetal well-being | No full text available |
| Roj | 2009 | Telemedical application for centralized home care of high-risk pregnancy based on control sharing approach | Wrong study design |
| Runkle | 2019 | Use of wearable sensors for pregnancy health and environmental monitoring: Descriptive findings from the perspective of patients and providers | Not home fetal monitoring |
| Ryu | 2021 | Comprehensive pregnancy monitoring with a network of wireless, soft, and flexible sensors in high-and low-resource health settings | Does not use cardiotocography |
| Santoni | 1986 | Trans-telephonic fetal cardiography in the monitoring of pregnancy | Unable to translate |
| Schramm | 2019 | Women's Attitudes Toward Self-Monitoring of Their Pregnancy Using Noninvasive Electronic Devices: Cross-Sectional Multicenter Study | Does not use cardiotocography |
| Schultes | 1991 | The monitoring of high-risk pregnancies by means of telephone cardiotocography | No full text available |
| Shindgikar | 2021 | 330 Evaluating the potential for home-based non-stress tests in the setting of COVID-19 pandemic | Wrong publication type |
| Shunsuke | 2021 | Preliminary report on the feasibility of remote CTG self monitoring at home with mobile device | Wrong publication type |
| Signorini | 2018 | Antepartum Fetal Monitoring through a Wearable System and a Mobile Application | Does not use cardiotocography |
| Signorini | 2020 | ICT4MOMs: An ICT Integrated Approach to Monitor and Manage Pregnancy Developmen | Wrong study design |
| Sipka | 2017 | Monitoring of Fetal Heart Rate via iPhone | Does not use cardiotocography |
| Su | 2002 | Clinical application of the expert type terminal of remote electronic fetal heart rate home monitoring system | Unable to translate |
| Sun | 2024 | NeuroFetalNet: Advancing Remote Electronic Fetal Monitoring with a New Dataset and Comparative Analysis of FHR and UCP Impact | Does not use cardiotocography |
| Takeuchi | 1980 | Telephone line transmission of fetal and maternal signals for remote monitoring | Not full text available |
| Tamsen | 1990 | Ambulatory fetal monitoring via telephone-experiences with a new system | Unable to translate |
| Tapia-Conyer | 2015 | Improving Perinatal Care in the Rural Regions Worldwide by Wireless Enabled Antepartum Fetal Monitoring: A Demonstration Project | Not home fetal monitoring |
| Thoulon | 1994 | [Our experience and evaluation trial of obstetrical telemonitoring] | Unable to translate |
| Torok | 1999 | Ten years' clinical experience with telemedicine in prenatal care in Hungary | Wrong publication type |
| Ubhi | 1995 | Modern fetal heart monitoring at home and in hospital | Wrong publication type |
| Uzan | 1989 | Home self-monitoring of at-risk pregnancies | Unable to translate |
| Valensise | 1991 | Telephone transmission of the cardiotocographic recording from the office to the obstetrical clinic | Unable to translate |
| vandenHeuvel | 2020 | Home-Based Monitoring and Telemonitoring of Complicated Pregnancies: Nationwide Cross-Sectional Survey of Current Practice in the Netherlands | Wrong study design |
| Vellacott | 1989 | Home monitoring of the fetus – an evaluation of patient-recorded traces | Wrong study design |
| Vermeulen-Giovagnoli | 2015 | The development of an obstetric tele-monitoring system | Not home fetal monitoring |
| Vintzileos | 1986 | Telephone transmission of fetal heart rate monitor data. The experience at the University of Connecticut Health Center | No full text available |
| Week | 1981 | Fetal heart rate monitoring using repeater telemetry methods | Not full text available |
| Xi | 2015 | Design of Smart Care Tele-Monitoring System for Mother and Fetus | Unable to translate |
| Xu | 2023 | Development of a portable home fetal heart rate monitor that connects to mobile phones | Not home fetal monitoring |
| Yang | 2021 | Application and clinical analysis of remote fetal heart rate monitoring platform in continuous fetal heart rate monitoring images | Not home fetal monitoring |
| Zach | 2011 | Mobile CTG - Fetal Heart Rate Assessment Using Android Platform | Wrong study design |
| Zhan | 2004 | Remote fetal monitoring for gestational diabetes mellitus | Unable to translate |
| Zang | 2018 | Design for Fetal Heartbeat Detection and Monitoring in Pregnancy Care | Not home fetal monitoring |
| Zuckerwar | 1993 | Development of a piezopolymer pressure sensor for a portable fetal heart rate monitor | Not home fetal monitoring |

AI, artificial intelligence; COVID, coronavirus disease; CTG, cardiotocography; FHR, fetal heartrate; ICT, information and communication technology.
