# Supplementary material for: Remote home cardiotocography: A systematic review and meta-analysis
Source: PLOS Digit Health. 2026 Jan 12;5(1):e0001184. doi: 10.1371/journal.pdig.0001184 (PMC12795381; doi:10.1371/journal.pdig.0001184)
Supplement: S5 Table — (DOCX) [file pdig.0001184.s005.docx]

**S5 Table:** Description of study methodology.

| **Title/year** | **Description of methodology** | **Study details** | **Operating environment** | **Receiving environment** | **Summary of findings** |  |  |  |  |  |
| --- | --- | --- | --- | --- | --- | --- | --- | --- | --- | --- |
| **Randomised controlled trial** | | | | | |  |  |  |  |  |
| Bekker 2023 (9) | Participants were randomly assigned to either hospital admission or telemonitoring in (1:1), stratified for the six diagnoses for inclusion and the six centres of inclusion, using block randomisation (block sizes of four and six). When assigned to telemonitoring, participants went home with devices for cardiotocography and blood pressure measurements and had daily contact with their care providers after digitally sending their home measurements. | **Sample size:** 201  **Gestation:** 2^nd^ and 3^rd^  **Recruitment location:** Netherlands  **High/Low risk patients:** High | **Environment:** Home  **Operator**: Patient  **Training:** Trained face-to-face to use the medical devices  **Technology:** Sense4Baby cardiotocography system (ICT Healthcare Technology Solutions) with online portal for remote review. | **Environment:** Hospital  **Receiver:** NR  **Training:** NR  **Technology:** Online portal to view CTG trace  **Communication:** Internet  **Transmission:** Synchronous  **Transmission delay:** NR | The primary outcome occurred in 31 (31%) of 100 participants in the telemonitoring group and in 40 (40%) of 100 participants in the hospital admission group. Adjusted for centre of inclusion, diagnosis, and nulliparity, the risk difference in primary outcome between both groups was 10·3% (95% CI –22·4 to 2·2) lower in the telemonitoring group, below the pre-defined non-inferiority margin of 10% absolute increase. |  |  |  |  |  |
| Birnie 1997 (30) | 150 consecutive women with high-risk pregnancies were randomised to either in-hospital or daily domiciliary monitoring. The main outcome measures were neonatal safety and cost-effectiveness. | **Sample size:** 150  **Gestation:** NR  **Recruitment location:** USA  **High/Low risk patients:** High | **Environment:** Home  **Operator**: Midwife  **Training:** NR  **Technology:** Sonicaid system 8000. Tracings transmitted by public telephone network to the hospital. | **Environment:** Hospital  **Receiver:** Obstetrician  **Training:** NR  **Technology:** NR  **Communication:** Telephone  **Transmission:** NR  **Transmission delay:** NR | Neonatal outcomes were equal. Domiciliary reduced mean antenatal costs from $3558($2841) to $1521 ($1459( per women (P<0.001). If costs varied by the addition of 50%, costs were still reduced. |  |  |  |  |  |
| Dawson 1999 (34) | Women in the control arm were intended to receive conventional care with standard midwifery visits. Women in the intervention arm received additional or longer visits and domiciliary fetal heartrate telemonitoring. | **Sample size:** 81  **Gestation:** NR  **Recruitment location:** UK  **High/Low risk patients:** High | **Environment:** Home  **Operator**: Patient or Midwife  **Training:** NR  **Technology:** Oxford Sonicaid 8000 system. CTG recordings were transmitted from the woman’s home by telephone using modems.  Community midwives took responsibility for domiciliary monitoring and transmission of the recordings to the base station. | **Environment:** Hospital  **Receiver:** NR  **Training:** NR  **Technology:** At the base station, a paper print-out was produced for analysis and inclusion in mothers’ records. Where there was doubt there was referral back to the consultant team.  **Communication:** Telephone  **Transmission:** Asynchronous  **Transmission delay:** NR | Eighty-one mothers were randomized. There were significant differences in midwifery intervention resources between domiciliary and control groups, with the former receiving a mean of 3.7 visits lasting 33.5min, compared with 1.4 visits lasting 12.8min for the latter. There were slightly more spontaneous labours and fewer Caesarean sections in the domiciliary group. Maternal satisfaction and anxiety were high in both groups. Domiciliary care increased the service costs by £21.02 per woman in terms of extra midwife travel and visiting time, and by a further £18.38 per woman in home monitoring equipment costs. This, however, was more than offset by health service savings from fewer clinic visits (£35.60) and fewer clinic ultrasound scans (£9.01). Adding the reductions in lost productivity to women and their partners (£34.51) suggests that domiciliary care was cheaper than conventional care, even if it did not greatly reduce inpatient days (a reduction nonetheless saving £184.24). |  |  |  |  |  |
| Dawson 1989 (35) | Sixty women were randomized 2: 1 for domiciliary surveillance or for conventional hospital care, with 30 and 17 records finally available for analysis. The groups were well matched for maternal, obstetric and socio-economic characteristics. | **Sample size:** 57  **Gestation:** NR  **Recruitment location:** UK  **High/Low risk patients:** High | **Environment:** Home  **Operator**: Midwife  **Training:** NR  **Technology:** NR | **Environment:** Hospital  **Receiver:** NR  **Training:** NR  **Technology:** NR  **Communication:** NR  **Transmission:** NR  **Transmission delay:** NR | In the domiciliary group, 21 (53%') of the women avoided hospital admission altogether, the admission rate was more than halved, and the mean proportion of the time spent in hospital was reduced from SO% to 16% of the observation period. The women who received domiciliary care were generally satisfied with the scheme. |  |  |  |  |  |
| Monincx 1997 (47) | 76 women were at random allocated to domiciliary care and 74 women to hospital care. Main Outcome Measures: Primary outcome measure was perinatal morbidity, measured by Prechtl’s neonatal neurological optimality score. Secondary outcome variables were the occurrence of complications, obstetric interventions at labour, birthweight, gestational age at delivery and maternal and neonatal admission rates. | **Sample size:** 150  **Gestation:** 3^rd^  **Recruitment location:** Netherlands  **High/Low risk patients:** High | **Environment:** Home  **Operator**: Midwife or physician  **Training:** NR  **Technology:** Oxford System 8000. Fetal heart rate was recorded for a minimum of 30 min and sent by modem to the hospital. | **Environment:** Hospital  **Receiver:** Obstetrician  **Training:** NR  **Technology:** NR  **Communication:** Telephone  **Transmission:** Synchronous  **Transmission delay:** NR | In both groups there was one case of perinatal mortality. In both treatment groups the median of the neurological optimality score was found at 59. Also, secondary outcome variables did not show significant differences. |  |  |  |  |  |
| Wang 2019 (59) | Women were randomly divided into two groups: the experimental group, which engaged in remote fetal monitoring, and the control group, which adopted traditional cardiac monitoring. In order to get more effective data, we used the Kalman filter and audio repair algorithms to preprocess the collected data. During follow-up observation, we compared the two groups using neonatal cardiac monitoring by employing the non-stress test and observed the occurrence of neonatal asphyxia. | **Sample size:** 160  **Gestation:** 3^rd^  **Recruitment location:** China  **High/Low risk patients:** NR | **Environment:** Home  **Operator**: NR  **Training:** NR  **Technology:** Portable intelligent medical terminal system features include the following: (1) multi-function portable hardware design. Together portable mobile medical box’s features are integrated electrocardiogram (ECG), blood pressure, blood oxygen, and related processing circuits. | **Environment:** Hospital  **Receiver:** Specialist  **Training:** NR  **Technology:** NR  **Communication:** Internet  **Transmission:** Synchronous  **Transmission delay:** NR | The incidence of neonatal abnormal non-stress test in the experimental group and the control group was 33.6% and 17.3%, respectively; the difference was statistically significant (p < 0.05). The incidence of neonatal asphyxia in the experimental group was 12.5%, which was significantly lower than in the control group (30%; p < 0.05). We have found that women in the late stage of pregnancy who adopted remote fetal monitoring could detect abnormal non-stress test earlier and thus increase in the detection of rate of neonatal asphyxia. |  |  |  |  |  |
| Zhou 2023 (60) | Pregnant women were randomly divided into two groups: the control group (n = 400), which was given traditional management, and the observation group (n = 400), which received remote monitoring technology on this basis. The two groups were compared with neonatal asphyxia, pregnancy outcomes, Edinburgh postnatal depression scale scores (EPDS), prenatal examination expenses and total time consumption | **Sample size:** 800  **Gestation:** 3^rd^  **Recruitment location:** China  **High/Low risk patients:** High | **Environment:** Home  **Operator**: Patient  **Training:** NR  **Technology:** Ultrasonic Doppler Electronic Fetal Monitoring (EFM) instrument. Then transmitted the measured data signals to the system’s tag-end server “cloud platform” through APP in smartphone. | **Environment:** Hospital  **Receiver:** Specialist  **Training:** NR  **Technology:** Tag-end server interpreted the monitoring results by auto intelligent (AI) and timely sent messages to notify the medical staff to review the data.  **Communication:** Internet  **Transmission:** Asynchronous  **Transmission delay:** NR | There were no statistically significant differences between the groups in pregnancy outcome and neonatal outcome (P > 0.05). However, total EPDS score of 12.5% pregnant women in the observation group were higher than 12. The traditional monitoring group had significantly higher mean EPDS scores compared with the remote monitoring group (7.79 ± 3.58 vs 5.10 ± 3.07; P < 0.05). The results showed a significant difference in maternity expenses (2949.83 ± 456.07 vs 2455.37 ± 506.67; P < 0.05) and total time consumption (42.81 ± 7.60 vs 20.43 ± 4.16; P < 0.05) between the groups. |  |  |  |  |  |
| **Feasibility study** | | | | | |  |  |  |  |  |
| Acker 1989 (28) | To describe a new device, the Genesis Fetal Monitor System (PDS, Inc.), that permits bioelectronic fetal assessment to be performed at home with immediate interpretation by skilled consultants. The system is designed to be used by nonprofessional’s; it is capable of accurately and rapidly transmitting a complete antenatal fetal monitor tracing over standard telephone lines and can rapidly duplicate a monitor strip for prompt evaluation. | **Sample size:** 12  **Gestation:** NR  **Recruitment location:** USA  **High/Low risk patients:** NR | **Environment:** Home  **Operator**: Initially obstetric nurse, then the patient  **Training:** Participants were trained in operating the necessary equipment for remote visits.  **Technology:** Genesis Fetal Monitor System, which includes a recorder, a communication module and a receiver system, accurately and rapidly transmits a complete antenatal fetal monitor tracing over standard telephone lines and duplicates the original tracing at the receiver center. | **Environment:** Hospital  **Receiver:** Consultant  **Training:** NR  **Technology:** The receiver system is operated by trained personnel and includes a modem, computer and software designed by PDS, Inc., to transfer and store received data, generate duplicate monitor tracings from the transmitted data, perform statistical analyses and prepare reports.  **Communication:** Telephone  **Transmission:** Asynchronous  **Transmission delay:** 2 minutes | In all cases in this study the original and generated tracings were superimposable. No significant technical problems were encountered. Bioelectronic fetal assessment can be performed from a nonhospital setting, including the patient's home, with immediate interpretation of the tracing by skilled consultants. |  |  |  |  |  |
| Axelrod 2025 (62) | Prospective study involving 20 women with gestational diabetes at ≥32 weeks of pregnancy, alternating between remote and in-clinic weekly visits. Remote assessments began with women self-measuring vital signs and using a digital urine dipstick. The remote encounter started with a midwife performing anamnesis and remotely connecting women to the fetal nonstress test. A physician concluded the meeting with remote sonographic assessment of amniotic fluid maximal vertical pocket that together with the nonstress test provided the modified biophysical assessment as well as a video encounter and ongoing glycaemic control assessment. We assessed the feasibility of remote visits, compared visit durations, evaluated women's satisfaction using the Telehealth Usability Questionnaire, examined glucose documentation adherence during hybrid care compared with the following period until birth, and assessed GD-related clinical outcomes. | **Sample size:** 20  **Gestation:** 3rd  **Recruitment location:** Isreal  **High/Low risk patients:** High | **Environment:** Home  **Operator**: Patient conducted through virtual communication with the healthcare provider.  **Training:** Prior to the first remote visit, all participants were trained by a midwife using the remote ultrasound probe, the remote fetal belt, the digital urine dipstick kit, and the digital gestational diabetic app.  **Technology:** All participants received a dedicated mobile device (Samsung Galaxy) equipped to support the various applications needed for the remote visits, as well as a cellular data plan, fetal monitor device (INVU by Nuvo), fetal ultrasound probe (Pulsenmore Ltd), blood pressure cuff, and two digital home urine test kits (Healthy.io Ltd). | **Environment:** Hospital  **Receiver:** A midwife observed real-time assessment of fetal monitoring, and a physician remotely assessed online fetal ultrasound.  **Training:** NR  **Technology:** Virtual communication with the healthcare provider (partly by video through the Datos Ltd. Platform.  **Communication:** Internet  **Transmission:** Synchronous  **Transmission delay:** NR | Remote visits had a success rate of 97.4% (38 of 39), with significantly shorter durations compared with in-clinic visits (median 59.0 min vs. 159.0 min, P< 0.001). Women expressed high satisfaction (6.6 of 7), and adherence with recording fasting glucose values during the study period was significantly higher than the following period until birth (92.2% vs. 61.8%, P= 0.001). Notably, none required induction of labour for glycaemic control imbalance, and there were no cases of macrosomia, shoulder dystocia, or neonatal hypoglycaemia. |  |  |  |  |  |
| Currie 1986 (32) | A simple method whereby the fetal heart rate can be monitored in hospital while the mother is sitting at home has been developed. This paper describes a feasibility study undertaken to assess the practicality of its use. | **Sample size:** 21  **Gestation:** 3^rd^  **Recruitment location:** UK  **High/Low risk patients:** Low | **Environment:** Home  **Operator**: Patient  **Training:** Each woman was taught how to find her baby's heartbeat using a Sonicaid D206 fetal heart detector and how to position a telephone handset over its loudspeaker.  **Technology:** Sonicaid D206 fetal heart detector. When the telephonic link with the hospital was established a check was made to ensure that the fetal heartbeat was coming through clearly before the recording was started. | **Environment:** Hospital  **Receiver:** NR  **Training:** NR  **Technology:** Once the fetal heart signal was being transmitted over the telephone lines and was being received in the hospital, it was amplified and fed into a Hewlett Packard 8040 CTG machine  **Communication:** Telephone  **Transmission:** Synchronous  **Transmission delay:** NR | The women learnt quickly and generally had no trouble in finding a clear fetal heartbeat. If the baby moved it was sometimes necessary to reangle the transducer slightly to maintain a clear heartbeat, but this was a skill which was acquired with practice. By the end of the first recording session most of the women were able to achieve good recordings. They all enjoyed the experience of listening to their baby's heart beating and indeed sometimes the whole family would join them. The cost of the Sonicaid D206's, the Fonadek amplifier and the communicating cable are all relatively low. In addition, all of these items have a long lifespan and need very little maintenance. The ordinary ward Hewlett Packard 8040 fetal heart monitor is very easy to adapt for home telemetry and no new monitor needs to be purchased. |  |  |  |  |  |
| Dalton 1986 (33) | Using the Sonicaid D206 fetal heart (FH) monitor at home with synchronous transmission for remote review. Paper to discuss feasibility within a low-risk patient cohort. | **Sample size:** 8  **Gestation:** 3^rd^  **Recruitment location:** UK  **High/Low risk patients:** Low | **Environment:** Home  **Operator**: Patient  **Training:** Sister then visits each patient at home, and teaches her to detect her own fetal heart  **Technology:** Domestic telephone is simply hung over the front of the Sonicaid D206 FH detector. The patient is instructed to check with the sister after 30 seconds of transmission, to ensure that the recording is proceeding satisfactorily. | **Environment:** Hospital  **Receiver:** Obstetrician  **Training:** NR  **Technology:** Telephone amplifier, which is directly linked to the telemetry input socket of a Hewlett Packard (HP) 8040 (option JO1) fetal heart monitor. The HP 8040 then prints out the telemetric fetal heart rate recording in real time.  **Communication:** Telephone  **Transmission:** Synchronous  **Transmission delay:** NR | High quality fetal home telemetry may be achieved without the help of a computer, using only a simple fetal heart detector at home, the public telephone lines and a conventional fetal heart rate monitor in the obstetric unit. Our system is called HOMEPLOT, and it is inexpensive to install, economical to maintain, simple to use and reliable in operation. |  |  |  |  |  |
| Dawson 1988 (36) | We report the development of a practical dedicated system for domiciliary fetal monitoring integrated into a scheme for its rational application. From experience of 1120 domiciliary recordings in 74 women (64 with high-risk pregnancies), we suggest that domiciliary monitoring applied within a structured clinical context should be as safe as monitoring in hospital. | **Sample size:** 74  **Gestation:** 2^nd^ & 3^rd^  **Recruitment location:** UK  **High/Low risk patients:** Low and high | **Environment:** Home  **Operator**: Patient or midwife  **Training:** NR  **Technology:** A small portable case containing a conventional widebeam ultrasound transducer, two controls, and rubber cups to accept the telephone handset when the 30-min recording is to be transmitted | **Environment:** Hospital  **Receiver:** NR  **Training:** NR  **Technology:** The finished recording is transmitted by telephone to the hospital for early review by prior arrangement, with one of us manning the central station.  **Communication:** Telephone  **Transmission:** Asynchronous  **Transmission delay:** 32.9 min (SD 47.37 min) | Of the 1120 recordings transmitted, 13 (1%) were considered to be uninterpretable. Sixteen were equivocal, and 25 were recognized as abnormal, that is, with absent high variation (5), absent reactive accelerations (4), decelerations (12), tachycardia with or without decelerations (2), and sinusoidal rhythm (2). In addition to the 1120 recordings transmitted, 11 were made but lost because of failed transmission |  |  |  |  |  |
| Feijen 1988 (37) | Here we describe a simpler method which also allows a reliable transmission of the fetal heart rate through the public telephone network. A cardiotocograph with autocorrelation system, a simple telephone amplifier, a telephone handset with adjustable speaker volume and an adapted portable ultrasonic fetal heart detector can form a simple but effective system for long distance telemetry of the fetal heart rate from patients’ homes using public telephone network. | **Sample size:** 3  **Gestation:** 3^rd^  **Recruitment location:** Netherlands  **High/Low risk patients:** High | **Environment:** Home  **Operator**: Patient  **Training:** NR  **Technology:** Sonicaid D102 portable ultrasonic fetal heart detector to detect the fetal heartbeat. By placing the microphone of the telephone’s handset against the detector’s loudspeaker, the fetal heart signal is fed into the telephone network. | **Environment:** Hospital  **Receiver:** NR  **Training:** NR  **Technology:** In the obstetric unit the fetal heart signal is conducted to the transducer of a cardiotocograph with an autocorrelation system (Hewlett Packard, 8040 A) through a simple telephone amplifier (Alecto, TA-150).  **Communication:** Telephone  **Transmission:** NR  **Transmission delay:** NR | More than 90% (43/47) were clearly interpretable. All the supervised telecardiograms (N = 27) had at least one trace of more than 20 min duration without signal loss of more than 20 s. The total signal loss in this group was only 4% of the total registration time of 763 min. In the unsupervised group the loss of signal was much greater, viz. 37% of a total registration time of 604 min. Nevertheless, 80% (16/20) of these tracing was still quite interpretable: no abnormalities were found. |  |  |  |  |  |
| Gonen 1990 (39) | We assessed the feasibility of introducing a home fetal heart rate monitoring system into a tertiary care perinatal unit. In the first stage of the study, 38 hospitalized high-risk pregnant patients recorded nonstress tests (NSTs) in their rooms in the hospital and transmitted the data to the receiving unit via the telephone. In the second stage, 34 women accomplished home monitoring in addition to the regular in hospital NSTs prescribed by their physicians. | **Sample size:** 34  **Gestation:** 3^rd^  **Recruitment location:** Canada  **High/Low risk patients:** NR | **Environment:** Home  **Operator**: Patient  **Training:** Patients were taught how to use the equipment with an average time of 8 minutes.  **Technology:** The home fetal monitor (Domiciliary Fetal Monitor; Huntleigh Technology Health Care Division, Cardiff, Wales, UK) consists of a small portable case containing a conventional wide-beam ultrasound transducer, an event marker, a simple touch-button control panel, and rubber cups to accept a telephone handset for transmission. | **Environment:** Hospital  **Receiver:** NR  **Training:** NR  **Technology:** The central station is IBM-compatible and uses a hard-wire modem. The resulting NST tracing (Figure 2) is 17 cm long (equivalent to 30 minutes of recording) and 4.7 cm high.  **Communication:** Telephone  **Transmission:** Synchronous  **Transmission delay:** NR | 72 patients, whose gestations ranged from 29-43 weeks, attempted 307 recordings, of which 93% were transmitted successfully. Ninety-eight percent of the received NSTs were considered interpretable. Based on our results, it seems that the use of the home fetal monitor by the patient in conjunction with all of the other components of a tertiary perinatal care center is feasible, reliable, and safe. |  |  |  |  |  |
| Gough 1986 (40) | We have developed a digital system for distant fetal heart rate recording with the facility of later rapid transmission by telephone into the hospital. A completed half-hour recording is transmitted to the central computer and permanently stored in less than 30 s, then immediately displayed for verbal reporting. | **Sample size:** 27  **Gestation:** 3^rd^  **Recruitment location:** UK  **High/Low risk patients:** Low | **Environment:** Home  **Operator**: NR  **Training:** NR  **Technology:** Data collection units each consist of a Doppler ultrasound detection device, filtering circuit, dedicated microprocessor board, and acoustic modem. These are secured in a small portable carrying case. The units usually operate on mains electricity, but they can also operate from an integral rechargeable battery pack | **Environment:** Hospital  **Receiver:** NR  **Training:** NR  **Technology:** A BBC Model B microcomputer in the hospital is used to control data transfer, permanent storage, and display.  **Communication:** Telephone  **Transmission:** Asynchronous  **Transmission delay:** 30 seconds | One hundred and seventeen recordings were made in 27 patients’ homes and of these 111 (95%) were easy to interpret. This expandable system meets the major functional requirements for practical and economical antenatal fetal heart rate monitoring at a distance. |  |  |  |  |  |
| Green 1992 (41) | A pilot trial was conducted to assess the technical feasibility of long-range fetal heart monitoring by telephone in an Australian setting. The indications for such monitoring and patient ability and attitude towards self-monitoring were also assessed. One hundred and fifty-seven tracings were received from 57 women using a simple doppler device to transmit fetal heart sounds to the central hospital fetal monitor. Thirty-three of the patients were in country hospitals and 24 were at home. Gestation ranged from 26 to 42 weeks’ gestation. | **Sample size:** 27  **Gestation:** 3^rd^  **Recruitment location:** Australia  **High/Low risk patients:** Low and high | **Environment:** Home  **Operator**: Patient  **Training:** NR  **Technology:** The home system used a portable fetal heart doppler (Sonicaid D206) from which the patient at home transmitted the signal via the public telephone system to the referral centre monitor. | **Environment:** Hospital  **Receiver:** NR  **Training:** NR  **Technology:** The central hospital monitor had a switching mechanism on the front panel which allowed transfer between normal operation and telemetry for either voice or fetal heart signal.  **Communication:** Telephone  **Transmission:** NR  **Transmission delay:** NR | Eighty six percent of the country’s hospital traces and 94% of the home traces were easily interpretable. Most traces were normal and appeared to encourage conservative management by the attendants. Two tracings were abnormal, and these influenced early delivery in both cases. Nearly all women using the monitor at home found the procedure easy and reassuring. Tracings from country hospitals were initiated usually after an acute antenatal complication, whereas the indications for home-monitoring were prompted by longer-term, medium risk factors. Home-monitoring may reduce the inconvenience and expense of inpatient or outpatient care and country hospitals without electronic fetal monitors may benefit from such a service. |  |  |  |  |  |
| Hamm 2023 (42) | This was a prospective, open-label, single site study of a wireless, remote pregnancy monitoring system (INVU by Nuvo Group, Ltd) in high-risk pregnancies to remotely perform clinically indicated NSTs instead of in clinic NSTs. | **Sample size:** 27  **Gestation:** 3^rd^  **Recruitment location:** USA  **High/Low risk patients:** High | **Environment:** Home  **Operator**: Patient  **Training:** NR  **Technology:** The INVU belt contains 8 biopotential and 4 acoustic sensors, which passively record abdominal signals. | **Environment:** Hospital  **Receiver:** NR  **Training:** NR  **Technology:** The monitoring system includes a Health Insurance Portability and Accountability Actecompliant mobile application that allows clinicians to view and interpret the NST and communicate with the patient in real time.  **Communication:** Internet  **Transmission:** Synchronous  **Transmission delay:** NR | INVU successfully obtained an interpretable NST in 93.9% of appointments, of which 98.3% were deemed reactive. Patients avoided an in-office visit in 88.5% of visits. Only 2 appointments (1.5%) resulted in a recommendation for nonurgent delivery, both for elevated blood pressure and neither related to the NST traces. 23 patients (79.3%) who attempted at least 1 remote NST completed the SUS, with a mean score of 76.5 of 100.0, indicating “good” usability, with 22 patients (95.7%) agreeing they would prefer remote NSTs vs in-office testing in a future pregnancy. |  |  |  |  |  |
| Horio 1998 (44) | We developed a telecommunication system for monitoring of the fetal heart rate and uterine contractibility at home or at local clinics, which could easily and securely screen large numbers of women. The design of the device and the results of an evaluation of this fetal monitor for home nonstress tests are described. | **Sample size:** 40  **Gestation:** NR  **Recruitment location:** Japan  **High/Low risk patients:** Low | **Environment:** Home  **Operator**: Patient  **Training:** NR  **Technology:** A fetal monitoring device developed for non-stress-test (NST) screening at home works on battery power, and is so small and lightweight (152 × 120 × 64 mm, 600 g) that a pregnant woman can monitor fetal Doppler ultrasound and record fetal heart rate (FHR) and uterine contraction (UC) data on an attached memory card at any time and in any place away from a hospital. | **Environment:** Hospital  **Receiver:** Physician  **Training:** NR  **Technology:** Evaluate data transmitted via public telephone lines, using a built-in modem in the monitor. UNIX operating system (OS) with an Ethernet connection to a personal computer for the medical doctor.  **Communication:** Telephone  **Transmission:** Asynchronous  **Transmission delay:** NR | The total number of NST data transmissions was 648, and the total amount of data received was more than 6.7 Mbytes. Of the 648 transmissions, 475 were adequate for clinical interpretation. Of the 101 failed NST data transmissions, 85 resulted from patient handling errors. However, 82.4% of these errors resulted in re-examination and transfer of new data by the patients, who were aware of the insufficiency of the original data. The main cause of noise in the data was zero-count data; this noise rate accounted for 4.1% of the data abnormalities. A questionnaire survey found that 96% of the participants wanted to use the monitor again in their next pregnancies, and 83% would recommend its use to pregnant friends. |  |  |  |  |  |
| Kerner 2004 (45) | Thirty-six women with high-risk pregnancies performed daily non-stress tests at home and transmitted the data to our perinatal care center by telephone. At each transmission, patients were asked by a physician about perceived fetal movements and uterine contractions and given the results. If the trace was unsatisfactory, further evaluation was performed. In addition, patients completed a questionnaire on quality of life and anxiety state before and after the study. | **Sample size:** 36  **Gestation:** 3rd  **Recruitment location:** Isreal  **High/Low risk patients:** High | **Environment:** Home  **Operator**: Patient  **Training:** Trained personnel instructed all patients in the use of the monitoring device.  **Technology:** Each patient was supplied with a lightweight ambulatory fetal-maternal cardiotocograph, CG900P. Data transmitted by phone. | **Environment:** Hospital  **Receiver:** NR  **Training:** NR  **Technology:** NR  **Communication:** Telephone  **Transmission:** NR  **Transmission delay:** NR | All patients were able to perform the tests and transmissions. The quality of recorded data was significantly correlated with maternal body mass index, but not with gestational age at the time of monitoring or birth weight. Thirty-nine of the total 562 traces (6.9%) were inconclusive or non-reassuring. After repeated testing, 32 of them (82%) were considered normal, and seven patients (18%) were referred for additional in-hospital evaluation. Of this group, four were discharged for further surveillance with routine home monitoring and the remaining three were hospitalized for continued evaluation. There were no significant immediate adverse maternal or neonatal outcomes because of the monitoring. Patient satisfaction was high. |  |  |  |  |  |
| Kitagawa 2000 (46) | We developed a telemedicine support system for pregnant women and evaluated it to see if that makes it possible 1) to manage pregnant women monitored at home in the same way as those who visit hospitals on an ambulatory basis, and 2) to prevent adverse events in women in a high-risk pregnancy. The findings obtained in the present study showed that this system is useful for both purposes. | **Sample size:** 22  **Gestation:** 2^nd^ & 3rd  **Recruitment location:** Japan  **High/Low risk patients:** Low & high | **Environment:** Home  **Operator**: Patient  **Training:** Received training with respect to the handling of the CTG probe.  **Technology:** The system developed incorporates a television telephone and a CTG monitor. | **Environment:** Hospital  **Receiver:** NR  **Training:** NR  **Technology:** Telecommunications were made every 2 weeks in order to receive data taken at home, including CTG monitoring data, and to conduct a clinical interview using a television telephone.  **Communication:** Telephone  **Transmission:** Asynchronous  **Transmission delay:** NR | The CTG data received from patients were comparable to those obtained at the hospital and could be fully used for diagnosis. Early diagnosis could be made, and complications could be prevented in 2 of the 17 high risk women. |  |  |  |  |  |
| Moore 1990 (48) | Domiciliary Fetal Monitoring is a new technique which allows fetal heart rate monitoring from within a patient’s home. The aims of the study were to assess: 1. The percentage of abnormal recordings in the first 100 patients who underwent domiciliary monitoring. 2. The obstetric and neonatal outcome of the study group, compared with the outcome of all patients booked for hospital confinement. 3. The changes in hospital admission rates and midwifery workload which occurred following the introduction of the new system. 4. Midwife and patient opinion of the DFM System. | **Sample size:** 100  **Gestation:** 3rd  **Recruitment location:** UK  **High/Low risk patients:** High | **Environment:** Home  **Operator**: Patient or midwife  **Training:** NR.  **Technology:** The Huntleigh Domiciliary Fetal Monitoring System was used. Eight portable ultrasound fetal heart rate (FHR) monitors were linked via telephone to a computer receiver | **Environment:** Hospital  **Receiver:** Obstetrician  **Training:** NR  **Technology:** Computer receiver, which was housed in the Delivery Suite.  **Communication:** Telephone  **Transmission:** Asynchronous  **Transmission delay:** The 30-minute cardiotocograph is transmitted by telephone, to a central labour ward computer, within 45 seconds. | Of 522 domiciliary cardiotocographs performed on 100 consecutive patients, 356 (68%) were performed for suspected intrauterine growth retardation or reduced fetal movements. Thirty-one (5.9%) recordings were abnormal; a repeat cardiotocograph in hospital was abnormal in 9 cases (1.3%); all such patients were delivered within 7 days. The induction rate for the study group was increased by a factor of 3.2 with respect to all hospital confinements; 49% of infants were small for gestational age (< 10th percentile). Caesarean section and forceps delivery rates were the same for both groups. No stillbirths occurred in the study group. After the introduction of domiciliary monitoring, the number and duration of hospital admissions for fetal surveillance were reduced by 29% and 52%, respectively. |  |  |  |  |  |
| Naef 1994 (49) | In this prospective study, ten women were tested using a Sonicaid TEAM portable monitor and a Hewlett Packard device in the health clinic. The women were then instructed on use of the Sonicaid device and were tested once a week in the home as well as in the high-risk clinic. All tests were reviewed independently by two of the authors to assess agreement in interpreting the NST. In the health clinic setting, a nonreactive NST was followed by a nipple stimulation contraction stress test (CST); in the home, a nonreactive NST was followed by maternal voice acoustic stimulation. | **Sample size:** 10  **Gestation:** NR  **Recruitment location:** USA  **High/Low risk patients:** High | **Environment:** Home  **Operator**: Patient  **Training:** NR.  **Technology:** Sonicaid (System 8000) TEAM portable monitor | **Environment:** Hospital  **Receiver:** NR  **Training:** NR  **Technology:** The tests from the home were transmitted compressed, via telephone, to a monitoring center, where the tracings were analysed to give feedback to the patient after transmission of the data.  **Communication:** Telephone  **Transmission:** Asynchronous  **Transmission delay:** NR | There was 100% correlation during the concurrent study. Two blinded authors agreed on all tests. During the second phase, eight of the ten subjects had NSTs that were consistently reactive in both the home and office settings. In one woman, a nonreactive NST in the clinic was followed by a negative CST. In the remaining patient, a reactive NST in the health clinic was followed 3 days later by a nonreactive NST in the home, which persisted in the provider's office. |  |  |  |  |  |
| Nakagawa 2020 (50) | This retrospective, single-institution study examined maternal telemedicine. The physicians remotely examined the pregnant women from their homes using a visual communication system which kept communication confidential, performed prenatal checkup and administered medical care according to their various blood pressures, weights and cardiotocograms. | **Sample size:** 44  **Gestation:** 2^nd^ & 3^rd^  **Recruitment location:** Japan  **High/Low risk patients:** NR | **Environment:** Home  **Operator**: Patient  **Training:** NR.  **Technology:** The cardiotocograph used was iCTG (Melody International Ltd.). The patient connects her smartphone or laptop to a computer at the Hospital via a visual communication system. | **Environment:** Hospital  **Receiver:** Physician  **Training:** NR  **Technology:** The patient connects her smartphone or laptop to a computer at the Hospital via a visual communication system. The visual communication system uses Kizuna Web (Borderless Vision Corp.) with a Transport Layer Security.  **Communication:** Internet  **Transmission:** Synchronous  **Transmission delay:** NR | Forty-four pregnant women received a total of 67 telemedicine interventions. Thirty-two pregnant women (73%) had complications, and 22 were primiparas (50%). Telemedicine interventions were provided 19 times at less than 26 weeks of gestation, 43 times between 26 and 36 weeks of gestation and 5 times after 37 weeks of gestation. There was one case with an abnormality diagnosed during the remote prenatal checkups, and the patient was hospitalized on the same day. However, there were no abnormal findings observed in mothers and children during the other 66 remote prenatal checkups and medical care. |  |  |  |  |  |
| Olesiak-Andryszczak 2025 (63) | This retrospective study included 32 women with high-risk pregnancies, who independently performed 581 examinations utilizing the portable cardiotocography device Pregnabit Pro (Nestmedic, Poland). Moreover, participants were asked to provide feedback on their experiences through a comprehensive survey. | **Sample size:** 32  **Gestation:** 3^rd^  **Recruitment location:** Poland  **High/Low risk patients:** high | **Environment:** Home  **Operator**: Patient  **Training:** Instructions provided by a midwife  **Technology:** Pregnabit Pro device, integrated with the Pregnabit Cloud telemedicine system. | **Environment:** Hospital  **Receiver:** Two qualified midwives and a specialist gynaecologist.  **Training:** NR  **Technology:** View of the monitoring the telemedicine Cloud system.  **Communication:** Internet  **Transmission:** Synchronous  **Transmission delay:** NR | In total, 95.7% of examinations successfully captured all necessary diagnostic data. Patients reported a high satisfaction rate, recognizing the potential of the home-based approach to improve their overall pregnancy experience. A preference for home-based testing over in-clinic visits was consistently noted among patients. |  |  |  |  |  |
| Romano 2009 (54) | Six selected patients, without effective pregnancy risk, were supplied with a lightweight ambulatory cardiotocograph equipped with two probes: a microphone and a pressure transducer. Monitoring covers the measurement of fetal heartbeat, uterine contractions and fetal movements. The selected patients were instructed by trained personnel in the use of the monitoring device. Patients were asked to perform the recording two times a week once and to transmit 20-min signal to the computer server of reference centre. The home monitoring sessions were performed in addition to the routine surveillance at the clinic; thus, the study monitoring did not replace clinic visits. | **Sample size:** 6  **Gestation:** 3^rd^  **Recruitment location:** Italy  **High/Low risk patients:** Low | **Environment:** Home  **Operator**: Patient  **Training:** Patients were instructed by trained personnel in the use of the monitoring device.  **Technology:** Lightweight ambulatory phonocardiotocograph. The fetal monitoring device sends pre-processed and compressed signals to a server through the GSM network. Recorded signals can also be displayed on a specially enabled mobile phone of the doctor examining the pregnant woman. | **Environment:** Hospital  **Receiver:** Physician  **Training:** NR  **Technology:** Gynaecologist could consult patient record stored on the computer server. The medical doctor could visualize the last sent recording, insert measure evaluation in the patient data record and send a text message to patient  **Communication:** Internet  **Transmission:** Asynchronous  **Transmission delay:** NR | Preliminary measurements on the selected pregnant women have shown that the use of this methodology significantly reduces the need of travel for patients and consequently their stress. Moreover, the obtained results, even if performed on normal fetuses, suggest that, after a short training, pregnant women can record and transmit long traces without troubles. Use of telemedicine system was generally well accepted by pregnant women increasing the possibility of fetal long-term home surveillance which in turn could increase the efficiency of the service offered to pregnant women. |  |  |  |  |  |
| Salomon 2004 (55) | This study was undertaken to audit a policy of fetal home monitoring (FHM) to achieve early detection of fetal heart rate (FHR) abnormalities in gastroschisis. | **Sample size:** 31  **Gestation:** 2^nd^ & 3^rd^  **Recruitment location:** France  **High/Low risk patients:** High | **Environment:** Home  **Operator**: NR  **Training:** NR  **Technology:** NR | **Environment:** Hospital  **Receiver:** NR  **Training:** NR  **Technology:** NR  **Communication:** Telephone  **Transmission:** NR  **Transmission delay:** NR | In 20 cases fetal heartrate monitoring remained normal. There were 16 elective caesarean sections, two emergency caesarean sections for fetal heartrate abnormalities detected by in-hospital monitoring, and two spontaneous premature vaginal deliveries. In 11 cases, abnormal monitoring was detected. There was one intrauterine death with acute ischemic necrosis of the large bowel. The other abnormalities consisted of decreased baseline variability with tachycardia (n = 7) or without tachycardia (n = 3) and were confirmed by in-hospital follow-up in nine cases, leading to emergency caesarean section. |  |  |  |  |  |
| Suemitsu 2023 (56) | Seventeen women diagnosed with FGR were enrolled. Patients performed iCTG for 1 hour twice daily to examine their fetuses; data were uploaded and saved on the cloud | **Sample size:** 17  **Gestation:** 3^rd^  **Recruitment location:** Japan  **High/Low risk patients:** High | **Environment:** Home  **Operator**: Patient  **Training:** Several training sessions were conducted to train the patients on using the device, and their understanding was confirmed.  **Technology:** iCTG is a portable, sleek, wireless, wearable, self-administered device comprising two units: a fetal heart and uterine contraction monitor and a tablet with built-in wireless communication features that send the FHR and uterine contraction data to a remote server, which is accessible via the internet. | **Environment:** Hospital  **Receiver:** NR  **Training:** NR  **Technology:** Medical information was obtained from the iCTG cloud server system and their medical records.  **Communication:** Internet  **Transmission:** Synchronous  **Transmission delay:** NR | The median and minimum compliance rates were 93.33 (interquartile range [IQR], 70.00–100.00) and 40.7, respectively. The median and minimum validity rates were 100.00 (IQR, 90.48–100.00) and 36.4, respectively. In this study, many of the patients were managed at home and underwent delivery as planned. However, three patients required emergency visits; one had a non-reassuring fetal status and underwent an emergency caesarean section. |  |  |  |  |  |
| Tamaru 2022 (57) | Using a mobile cardiotocogram device (iCTG, Melody International Ltd., Kagawa, Japan), participants of more than 34 gestational weeks measured the FHR by themselves at least once a week until hospitalization for delivery. We evaluated the acquisition rate of evaluable FHR recordings and the frequency of abnormal FHR patterns according to the CTG classification system of the Japan Society of Obstetrics and Gynecology (JSOG). The participants also underwent a questionnaire survey after delivery to evaluate their satisfaction level of self-monitoring FHR using the mobile CTG device. | **Sample size:** 101  **Gestation:** 3^rd^  **Recruitment location:** Japan  **High/Low risk patients:** NR | **Environment:** Home  **Operator**: Patient  **Training:** Healthcare professionals such as obstetricians or midwives taught and guided the participants on how to use the iCTG  **Technology:** The collected iCTG data could be seen by the participants on their own tablet in real time and were also stored in a cloud server via the Internet. | **Environment:** Hospital  **Receiver:** NR  **Training:** NR  **Technology:** Medical information was obtained from the iCTG cloud server system and their medical records.  **Communication:** Internet  **Transmission:** Synchronous  **Transmission delay:** NR | A total of 1278 FHR recordings from 101 women were analysed. Among them, 1276 (99.8%) were readable for more than 10 min continuously, and the median percentage of the total readable period in each recording was 98.9% (range, 51.4–100). According to the JSOG classification system, 1245 (97.6%), 9 (0.7%), 18 (1.4%), and four (0.3%) FHR patterns were classified as levels 1, 2, 3, and 4, respectively. The questionnaire survey revealed high participant satisfaction with FHR self-monitoring using the iCTG. |  |  |  |  |  |
| Uzan 1989 (58) | A simple system of recording the fetal heart rhythm that can be used by the patient herself at home. The recordings are transmitted daily or twice daily by telephone. The midwife in the maternity unit can look at the tracing and ask the patient to come if the tracing is insufficient or suspicious. | **Sample size:** 402  **Gestation:** 3^rd^  **Recruitment location:** France  **High/Low risk patients:** High and low | **Environment:** Home  **Operator**: Patient or midwife  **Training:** NR  **Technology:** A recording case: which includes a heartbeat sensor that the patient places on her abdomen. It also contains an event marker used to indicate active movements or uterine contractions perceived by the mother.  In the center of the case, two circular receptacles are provided to hold the telephone handset and thus transmit the information. | **Environment:** Hospital  **Receiver:** Midwife  **Training:** NR  **Technology:** On the other side of the telephone line, there is a standard computer with all the standard functions of a microcomputer. It is connected to a screen and a printer.  **Communication:** Telephone  **Transmission:** Asynchronous  **Transmission delay:** 45 seconds | The tracings correspond completely to those taken at the same time by the usual machines. Their interpretation does not give rise to any problems as compared with those of usual tracings. About 80% of the traces could be interpreted. They vary according to how far the pregnancy has progressed (73% at 32 weeks of pregnancy and 84% at term). Telephone transmission was carried out in 84% of cases. |  |  |  |  |  |
| Zizzo 2022 (61) | Remote self-monitoring was performed by pregnant women and included C-reactive protein, non-stress test by cardiotocography, temperature, blood pressure, heart rate, and a questionnaire concerning maternal and fetal wellbeing. Data was transferred to the hospital using a mobile device platform and evaluated by healthcare professionals. In case of non-reassuring registrations, the pregnant women were invited for assessment at the hospital. Primary outcome was perinatal death. Secondary outcomes were other maternal and perinatal complications. | **Sample size:** 400  **Gestation:** NR  **Recruitment location:** Denmark  **High/Low risk patients:** High | **Environment:** Home  **Operator**: Patient  **Training:** NR  **Technology:** Self-monitored data on maternal and fetal well-being were transferred electronically from the woman to the hospital (remote self-monitoring) with a mobile device including a telemedicine platform as the primary source for communication and data transfer. | **Environment:** Hospital  **Receiver:** NR  **Training:** Staff training workshops  **Technology:** Daily evaluation by healthcare professionals was based on information transferred digitally to the hospital by the OpenTele system.  **Communication:** Internet  **Transmission:** NR  **Transmission delay:** NR | No severe maternal complications were observed. Nine fetal or neonatal deaths occurred, all secondary to malformations, severe fetal growth restriction, extreme prematurity or lung hypoplasia in cases of premature prelabour rupture of membranes before 24 weeks. Even in the latter group, fetal and neonatal survival was 78% (18/23) and rose to 97% (60/62) when premature prelabour rupture of membranes occurred after a gestational age 23+6 weeks. None of the fetal or neonatal deaths were attributable to the home-management setting. |  |  |  |  |  |
| **Diagnostic accuracy study** | | | | | |  |  |  |  | **Domain 2: Study Methodology** |
| Porter 2021 (52) | Fetal heart rate recordings were performed using both the heartbeat monitor and cardiotocography to evaluate accuracy. Clinicians used the heartbeat monitor in the clinic. Women used the device, unassisted, during a clinic visit or at home. Obstetricians assessed the clinical utility of FHR traces. Women rated the heartbeat monitor using the System Usability Scale. | **Sample size:** 26  **Gestation:** 2^nd^ & 3^rd^  **Recruitment location:** Australia  **High/Low risk patients:** NR | **Environment:** Home  **Operator**: Patient  **Training:** A research nurse showed participants how to use the heartbeat monitor during a 5-minute training session.  **Technology:** The heartbeat monitor system includes a smartphone based interface that guides device placement and displays the FHR trace and calculated parameters (average FHR and maternal heart rate using beat-to-beat calculation, duration of FHR trace, duration of search time, and longest continuous FHR segment) on a Bluetooth-connected smartphone. | **Environment:** Hospital  **Receiver:** NR  **Training:** NR  **Technology:** NR  **Communication:** Internet  **Transmission:** NR  **Transmission delay:** NR | The accuracy of the heartbeat monitor was excellent compared with cardiotocography, with limits of agreement (95%) for mean FHR between 21.6 (CI 22.0 to 1.3) and +1.0 (CI 0.7–1.4) beats per minute (bpm), mean difference 20.3 bpm, intraclass coefficient 0.99. The FHR was detected on all occasions. Clinicians took a median (interquartile range) of 0.5 (0.2–1.2) minutes to detect the FHR, obtaining a continuous trace of longer than 1 minute in 95% (39/41) of occasions. Home users took a median of 0.5 (0.2–2.0) minutes to detect the FHR, obtaining a continuous trace of longer than 1 minute in 92% (24/26) of occasions, with a median total trace time of 4.6 (4.4–4.8) minutes. The traces were deemed clinically useful in 100% (55/55) of clinician and 97% (31/32) of home recordings. The heartbeat monitor ranked in the 96–100th percentile for usability and learnability. |  |  |  |  |  |
| Porter 2022 (53) | We compared the accuracy, clinical interpretability, and user experience of a patient-administered, wireless, fetal heartbeat monitor (HBM) designed for home use, to CTG. Initially, participants had paired HBM and CTG examinations performed in the clinic. Women then used the HBM unsupervised and rated the experience. | **Sample size:** 34  **Gestation:** 2^nd^ & 3^rd^  **Recruitment location:** Australia  **High/Low risk patients:** NR | **Environment:** Home  **Operator**: Patient  **Training:** A research nurse showed participants how to use the heartbeat monitor during a 5-minute training session.  **Technology:** The system includes a smartphone-based interface that displays the FHR trace and calculated parameters (average FHR and MHR using beat-to-beat calculation, duration of FHR trace, duration of search time, and longest continuous FHR segment) on a Bluetooth-connected smartphone and then uploads it to a clinical management system connected to the clinic. | **Environment:** Hospital  **Receiver:** Clinic staff and Senior obstetrician assessed clinical interpretability  **Training:** NR  **Technology:** Data uploaded to the clinical management system connected to the clinic for remote review.  **Communication:** Internet  **Transmission:** Synchronous  **Transmission delay:** NR | The accuracy of the HBM was excellent, with limits of agreement (95%) for mean fetal heart rate (FHR) between 0.72 and −1.78 beats per minute. The FHR was detected on all occasions and confirmed to be different from the maternal heart rate. Both methods were equally interpretable by obstetricians and had similar signal loss ratios. Thirty-four (100%) women successfully detected the FHR and obtained clinically useful cardiographic data using the device at home unsupervised. They achieved the required length of recording required for non-stress test analysis. The monitor ranked in the 96–100th percentile for usability and learnability. The HBM is as accurate as gold-standard CTG and provides equivalent clinical information. |  |  |  |  |  |
| **Pilot study** | | | | | |  |  |  |  |  |
| Gan 2023 (38) | Pregnant women used a mobile cardiotocogram device to measure the FHR at least once a week until delivery in the remote group. For the control group, pregnant women underwent traditional FHR monitoring once a week in the outpatient clinic. The rate of caesarean section, risk of postpartum haemorrhage and adverse neonatal outcomes were compared between the two groups. All the pregnant women completed a questionnaire survey to evaluate their acquisition of remote FHR self-monitoring. | **Sample size:** 1067  **Gestation:** Third  **Recruitment location:** China  **High/Low risk patients:** High and low | **Environment:** Home  **Operator**: Patient  **Training:** NR  **Technology:** Wireless remote FHR self-monitoring | **Environment:** Hospital  **Receiver:** NR  **Training:** NR  **Technology:** NR  **Communication:** Internet  **Transmission:** NR  **Transmission delay:** NR | The women in the remote FHR monitoring group were more likely to be nulliparous (P<0.001), more likely to have a higher education level (P =0.003). There was no significant difference in the risk of caesarean section (P=0.068) or postpartum haemorrhage (P=0.836) between the two groups. No difference in fetal complications was observed across groups, with the exception of the incidence of NICU stays, which was higher in the remote group (12.0% vs. 8.3%, P=0.044). The questionnaire survey showed that the interval time (P=0.001) and cost (P=0.010) of fetal heart rate monitoring were lower in the remote group. Regarding age, pre-pregnancy BMI, risk factors, education level, maternal risk and household income, senior high school (OR 2.86, 95% CI 1.67–4.90, P<0.001, undergraduate (OR 2.96, 95% CI 1.73–5.06, P<0.001, advanced maternal age (OR 1.42, 95% CI 1.07–1.89, P=0.015) and high-risk pregnancy (OR 1.61, 95% CI 1.11–2.35, P=0.013) were independent factors for pregnant women to choose remote fetal monitoring. |  |  |  |  |  |
| Pan 2024 (51) | 800 utilized remote monitoring, with 760 of them completing the self-rating anxiety scale (SAS) and self-rating depression scale (SDS) assessments using the devices for 1 month. The control group comprised 2100 pregnant women who did not use remote monitoring. Additionally, 80 pregnant women concurrently employed both REFM and traditional electronic fetal monitoring, and their respective curve coincidence rates were determined through curve fitting. Primary outcomes encompassed pregnancy outcomes in both groups, average curve coincidence rates between remote and traditional monitoring, as well as SDS and SAS scores. | **Sample size:** 2860  **Gestation:** Third  **Recruitment location:** China  **High/Low risk patients:** High and low | **Environment:** Home  **Operator**: Patient  **Training:** NR  **Technology:** System enabled remote monitoring via wireless internet connectivity from the fetal monitoring center, accessible to pregnant women through mobile internet | **Environment:** Hospital  **Receiver:** NR  **Training:** NR  **Technology:** Online medical professional's system (Information Analysis Center)  **Communication:** Internet  **Transmission:** Synchronous  **Transmission delay:** NR | Among the 760 pregnant women who completed SAS and SDS assessments, their average SAS scores before and after 1 month of remote monitoring usage were 43.09 ± 8.04 and 41.58 ± 6.59, respectively. Concurrently, the average SDS scores before and after 1 month of remote monitoring usage were 45.45 ± 9.60 and 44.80 ± 9.17, respectively. A statistically significant decrease was observed in SAS scores (P= 0.005), whereas no significant difference was noted in SDS scores (P= 0.340). Furthermore, a statistically significant difference in the rate of adverse pregnancy outcomes (neonatal asphyxia) emerged between the two groups, those who employed remote monitoring and those who did not (P= 0.021). In the subset of 80 pregnant women employing both remote and traditional monitoring, all 80 results showed precise congruence between the two methods. The average coincidence rate was determined to be 79.45% ± 12.64%. |  |  |  |  |  |
| **Valuation study** | | | | | |  |  |  |  |  |
| Birnie 2000 (31) | Each woman was asked to assign two valuations by putting a mark on each of two uncalibrated 100 mm visual analogue scales (VAS). The left side anchor was labelled “valued extremely negative,” the right one was labelled “valued extremely positive.” The first valuation referred to the allocated monitoring strategy (i.e., the strategy that the women had actually experienced). The second valuation referred to the alternative, not allocated, monitoring strategy. The hypothetical nature of the valuation assigned to the alternative strategy was acknowledged in the phrasing of the question. Both visual analogue scales were identical in all other respects. | **Sample size:** 90  **Gestation:** NR  **Recruitment location:** Netherlands  **High/Low risk patients:** High | **Environment:** Home  **Operator**: Midwife  **Training:** NR  **Technology:** Women allocated to domiciliary monitoring were monitored daily at their homes by a midwife using portable equipment. | **Environment:** Hospital  **Receiver:** NR  **Training:** NR  **Technology:** NR  **Communication:** NR  **Transmission:** NR  **Transmission delay:** NR | Valuations were expressed as a between-subject difference (assigned by the women allocated to the respective strategies) and as within-subject differences (assigned by all women). Domiciliary monitoring was valued higher by the women allocated to that strategy (P = 0.10). In-hospital monitoring was valued higher by the women allocated to that strategy (P = 0.02). The average within-subject differences differed by allocated strategy (P = 0.01). The within-subject valuation differences showed large variability between and within groups. An overrepresentation of women favouring domiciliary monitoring and asymmetric treatment experience inflated the average within-subject difference in the domiciliary group but deflated that difference in the in-hospital group. Neither the average between-subject difference nor the average within subject differences are free of bias. |  |  |  |  |  |
| **Qualitative study** | | | | | |  |  |  |  |  |
| Aasbo 2024 (27) | To address diverse perspectives regarding home based telemonitoring (HBTM) of high-risk pregnancies, four different groups of experienced healthcare providers or users were interviewed (n= 21). Focus group interviews were conducted separately with midwives, obstetricians, and women who had previously experienced stillbirth. Six individual interviews were conducted with hospitalized women with ongoing high-risk pregnancies, representing potential candidates for HBTM. None of the participants had any previous experience with HBTM of pregnancies. The study is embedded in a social constructivist research paradigm. Interviews were analysed using a thematic approach. | **Sample size:** 21  **Gestation:** NR  **Recruitment location:** Norway  **High/Low risk patients:** High | **Environment:** Home  **Operator**: Patient  **Training:** NR  **Technology:** Tablet connected with a device for highly reliable, remote CTG monitoring, as well as equipment to measure blood pressure, temperature, and C-reactive protein (the latter relevant for the premature prelabour rupture of membranes pregnancies). Patients download a smartphone application to report parameters for in-clinic assessment. | **Environment:** Hospital  **Receiver:** Obstetrician  **Training:** NR  **Technology:** NR  **Communication:** Internet  **Transmission:** NR  **Transmission delay:** NR | Participants acknowledged the benefits and potentials of more active roles for both care recipients and providers in HBTM. Concerns were clearly addressed and articulated in the following themes: eligibility and ability of women, availability of midwives and obstetricians, empowerment and patient safety, and shared responsibility. All groups problematized issues crucial to maintaining a sense of safety for care recipients, and healthcare providers also addressed issues related to maintaining a sense of safety also for the care providers. Conditions for HBTM were understood in terms of optimal personalized training, individual assessment of eligibility, and empowerment of an active patient role. These conditions were linked to the importance of competent and experienced midwives and obstetricians operating the monitoring, as well as the availability and continuity of care provision. |  |  |  |  |  |
| Bendix 2024 (29) | A qualitative, semi-structured interview study was conducted with women with ongoing experience in performing home-based telemonitoring procedures for high-risk pregnancy complications. Purposeful sampling strategy and data saturation were applied followed by verbatim transcription. The data were analysed using systematic text condensation. | **Sample size:** 15  **Gestation:** NR  **Recruitment location:** Denmark  **High/Low risk patients:** High | **Environment:** Home  **Operator**: Patient  **Training:** A thorough training session is conducted by a tele-midwife  **Technology:** Android tablet for submission of questionnaire answers, values, and results, and a fetal CTG monitor. | **Environment:** Hospital  **Receiver:** Tele-midwife and/or obstetrician  **Training:** NR  **Technology:** NR  **Communication:** Internet  **Transmission:** Asynchronous  **Transmission delay:** NR | Fifteen informants participated in the study and four major themes emerged. The study revealed that performing telemonitoring was overall positively experienced as an ‘Empowering yet challenging responsibility’ as well as an ‘Extended patient-clinician partnership.’ There were pros and cons as to the influence of telemonitoring in everyday life; ‘Tele-comfort yet ambivalence’ and that it could be accompanied by annoying practical issues; ‘Accompanying remote issues.’ |  |  |  |  |  |
| Hamm 2024 (43) | After 2 attempted remote sessions, cohort patients were invited to participate in a semi-structured qualitative interview until thematic saturation was achieved. A Consolidated Framework for Implementation Research influenced interview guide elicited information on (1) experiences with in-clinic and remote monitoring, (2) barriers to or facilitators of remote monitoring, and (3) suggestions for improvement. For the analysis, an integrated approach was used in NVivo with excellent intercoder reliability | **Sample size:** 12  **Gestation:** 3^rd^  **Recruitment location:** USA  **High/Low risk patients:** High | **Environment:** Home  **Operator**: Patient  **Training:** NR  **Technology:** INVU device to perform remote non-stress tests. | **Environment:** Hospital  **Receiver:** NR  **Training:** NR  **Technology:** NR  **Communication:** NR  **Transmission:** NR  **Transmission delay:** NR | Few participants expressed positive experiences with in-clinic sessions, including a sense of ease or comfort. However, the majority spoke about coordinating childcare and frustrations surrounding travel time, public transportation, work scheduling, and parking with each factor perceived as being financially impactful. The majority reported excitement for at-home monitoring, stating a belief that it would be convenient and less costly. Participants reported feeling relieved of the logistics and expenses of in-clinic monitoring. Concerns with remote monitoring were primarily hypothetical what-ifs. Yet, when probed, the majority reported reassurance about receiving similar monitoring as received in-clinic. Some participants mentioned the need for increased set-up support. One mentioned difficulty with staying still during the experience. For facilitators, in addition to the decreased need for childcare, transportation, and cost, several stated they appreciated clinician communication through the application. |  |  |  |  |  |
| VanDenHeuvel 2020 (11) | Using secured Facebook Groups, four online focus groups: two focus groups with women who were admitted during pregnancy (n = 11) and two with women who received home telemonitoring in the pilot phase (n = 11) were created. The qualitative data was analysed thematically. | **Sample size:** 22  **Gestation:** NR  **Recruitment location:** Netherlands  **High/Low risk patients:** High | **Environment:** Home  **Operator**: Patient  **Training: I**nstructions provided by a member of our centre’s Obstetric Telemonitoring Team (consisting of a clinical midwife, the resident on ward supervised by an obstetrician)  **Technology:** Wireless devices for blood pressure (Microlife WatchBP) and cardiotocography (Sense4Baby, BMA- Telenatal, The Netherlands) were used for daily follow up of patients. | **Environment:** Hospital  **Receiver:** Obstetric Telemonitoring Team  **Training:** NR  **Technology:** NR  **Communication:** Internet  **Transmission:** Asynchronous  **Transmission delay:** NR | Four major themes emerged from both participant groups: [1] care experience, [2] emotions regarding pregnancy, [3] privacy and [4] impact on daily life. Different views were reported on all four themes, resulting in a direct comparison of experiences during hospitalization and telemonitoring. Most admitted patients reported a growing sense of boredom and anxiety during their clinical admission. Lack of privacy in the ward was a great concern, as it affected their contact with hospital staff and family. This issue was not reported amongst telemonitored women. These participants still felt like a patient at times but responded that the comfort of their own home and bed was pleasant. Only a minority of telemonitored participants reported being anxious at times at home, while not having a physician or nurse nearby. Being at home resulted in less travel time for partners or family for hospital visits, which had positive effects on family life. |  |  |  |  |  |

CI, confidence interval; CTG, cardiotocography; FHR, fetal heartrate; NICU, neonatal intensive care unit; NR, not recorded; NST, non-stress test; OR, odds ratio; UK, United Kingdom; USA, United States of America.
