# Supplementary material for: Remote home cardiotocography: A systematic review and meta-analysis
Source: PLOS Digit Health. 2026 Jan 12;5(1):e0001184. doi: 10.1371/journal.pdig.0001184 (PMC12795381; doi:10.1371/journal.pdig.0001184)
Supplement: S6 Table — (DOCX) [file pdig.0001184.s006.docx]

| **Observational studies** | | | | | | | | | | | | | | | | | | | | | | | | | | | | | | | | | | |
| --- | --- | --- | --- | --- | --- | --- | --- | --- | --- | --- | --- | --- | --- | --- | --- | --- | --- | --- | --- | --- | --- | --- | --- | --- | --- | --- | --- | --- | --- | --- | --- | --- | --- | --- |
| **Domain 1: Generic quality standards** | | | | | | | | | | | | | | | | | | | | | | | | | | | | | | | | | | |
|  | | Acker 1989 | Axelrod 2025 | Birnie 2000 | Currie 1986 | Dalton 1986 | | Dawson 1988 | Feijen 1988 | Gan 2023 | Gonen 1990 | Gough 1986 | | Green 1992 | Hamm 2023 | Horio 1988 | Kerner 2004 | Kitagawa 2000 | | Moore 1990 | Naef 1994 | Nakagawa 2020 | Olesiak-Andryszczak 2025 | Pan 2024 | | Porter 2021 | Porter 2022 | Romano 2009 | Salomon 2004 | Suemitsu 2023 | | Tamaru 2022 | Uzan 1989 | Zizzo 2022 |
| 1. Focused question | | 1 | 1 | 1 | 1 | 1 | | 1 | 1 | 1 | 1 | 1 | | 1 | 1 | 1 | 1 | 1 | | 1 | 1 | 1 | 1 | 1 | | 1 | 1 | 1 | 1 | 1 | | 1 | 1 | 1 |
| 1. Study design appropriate | | 2 | 1 | 3 | 1 | 1 | | 1 | 1 | 1 | 1 | 1 | | 1 | 1 | 1 | 1 | 1 | | 1 | 1 | 1 | 1 | 1 | | 1 | 1 | 1 | 1 | 1 | | 1 | 1 | 1 |
| 1. Size appropriate? | | 2 | 2 | 2 | 2 | 2 | | 1 | 3 | 1 | 2 | 2 | | 2 | 2 | 2 | 2 | 2 | | 2 | 3 | 2 | 2 | 1 | | 2 | 2 | 3 | 2 | 2 | | 2 | 2 | 2 |
| 1. Conflict of interest | | 3 | 1 | 3 | 3 | 3 | | 3 | 3 | 1 | 3 | 3 | | 3 | 1 | 3 | 2 | 2 | | 3 | 2 | 1 | 1 | 1 | | 1 | 1 | 3 | 3 | 1 | | 1 | 2 | 1 |
| 1. Ethical approval | | 3 | 1 | 3 | 3 | 3 | | 3 | 3 | 1 | 3 | 3 | | 1 | 1 | 3 | 1 | 3 | | 3 | 3 | 1 | 1 | 1 | | 1 | 1 | 3 | 3 | 1 | | 1 | 3 | 1 |
| 1. Informed consent | | 2 | 1 | 1 | 3 | 2 | | 3 | 3 | 1 | 3 | 3 | | 2 | 1 | 3 | 1 | 1 | | 3 | 3 | 1 | 1 | 1 | | 1 | 1 | 1 | 3 | 1 | | 1 | 3 | 1 |
| 1. Replicable methods | | 3 | 1 | 1 | 3 | 2 | | 1 | 3 | 2 | 1 | 2 | | 1 | 3 | 1 | 1 | 3 | | 1 | 3 | 3 | 1 | 2 | | 1 | 1 | 1 | 3 | 1 | | 3 | 1 | 3 |
| 1. Selective reporting | | 2 | 1 | 1 | 1 | 1 | | 1 | 1 | 1 | 1 | 1 | | 1 | 1 | 1 | 1 | 1 | | 1 | 1 | 1 | 1 | 1 | | 1 | 1 | 1 | 1 | 1 | | 1 | 1 | 1 |
| 1. Study limitations | | 1 | 1 | 1 | 3 | 1 | | 3 | 3 | 1 | 3 | 3 | | 1 | 1 | 2 | 2 | 1 | | 3 | 3 | 3 | 1 | 1 | | 1 | 1 | 2 | 1 | 1 | | 1 | 2 | 1 |
| 1. Generalisability reported | | 2 | 1 | 1 | 3 | 1 | | 1 | 2 | 1 | 2 | 3 | | 1 | 2 | 2 | 1 | 3 | | 2 | 1 | 1 | 1 | 1 | | 1 | 1 | 1 | 1 | 1 | | 1 | 1 | 1 |
| **Domain 2: Patient/Participant selection** | | | | | | | | | | | | | | | | | | | | | | | | | | | | | | | | | | |
|  | | Acker 1989 | Axelrod 2025 | Birnie 2000 | Currie 1986 | Dalton 1986 | | Dawson 1988 | Feijen 1988 | Gan 2023 | Gonen 1990 | Gough 1986 | | Green 1992 | Hamm 2023 | Horio 1988 | Kerner 2004 | Kitagawa 2000 | | Moore 1990 | Naef 1994 | Nakagawa 2020 | Olesiak-Andryszczak 2025 | Pan 2024 | | Porter 2021 | Porter 2022 | Romano 2009 | Salomon 2004 | Suemitsu 2023 | | Tamaru 2022 | Uzan 1989 | Zizzo 2022 |
| 1. Simulation representative | | 4 | 4 | 4 | 4 | 4 | | 4 | 4 | 4 | 4 | 4 | | 4 | 4 | 4 | 4 | 4 | | 4 | 4 | 4 | 4 | 4 | | 4 | 4 | 4 | 4 | 4 | | 4 | 4 | 4 |
| 1. Level of expertise described | | 3 | 3 | 3 | 3 | 3 | | 3 | 3 | 3 | 3 | 3 | | 3 | 3 | 3 | 1 | 3 | | 1 | 3 | 1 | 3 | 1 | | 2 | 2 | 1 | 3 | 3 | | 3 | 3 | 3 |
| 1. Methods of participation | | 2 | 1 | 1 | 1 | 1 | | 1 | 3 | 1 | 1 | 2 | | 2 | 1 | 1 | 1 | 1 | | 1 | 2 | 1 | 1 | 1 | | 1 | 1 | 1 | 1 | 2 | | 1 | 1 | 1 |
| 1. Inappropriate exclusions | | 2 | 1 | 1 | 2 | 3 | | 1 | 2 | 1 | 2 | 3 | | 3 | 1 | 3 | 1 | 2 | | 2 | 3 | 1 | 1 | 1 | | 1 | 1 | 3 | 3 | 3 | | 1 | 2 | 1 |
| 1. Participant selection | | 3 | 1 | 2 | 2 | 2 | | 1 | 3 | 1 | 1 | 3 | | 2 | 1 | 2 | 1 | 2 | | 2 | 2 | 1 | 1 | 1 | | 1 | 1 | 2 | 2 | 2 | | 1 | 2 | 1 |
| **Domain 3: Index test(s)** | | | | | | | | | | | | | | | | | | | | | | | | | | | | | | | | | | |
|  | | Acker 1989 | Axelrod 2025 | Birnie 2000 | Currie 1986 | Dalton 1986 | | Dawson 1988 | Feijen 1988 | Gan 2023 | Gonen 1990 | Gough 1986 | | Green 1992 | Hamm 2023 | Horio 1988 | Kerner 2004 | Kitagawa 2000 | | Moore 1990 | Naef 1994 | Nakagawa 2020 | Olesiak-Andryszczak 2025 | Pan 2024 | | Porter 2021 | Porter 2022 | Romano 2009 | Salomon 2004 | Suemitsu 2023 | | Tamaru 2022 | Uzan 1989 | Zizzo 2022 |
| 1. Reviewers blinded | | 2 | 3 | 3 | 3 | 3 | | 3 | 3 | 2 | 3 | 3 | | 3 | 3 | 3 | 3 | 3 | | 3 | 1 | 3 | 3 | 3 | | 3 | 3 | 3 | 3 | 3 | | 3 | 3 | 3 |
| 1. Outcome measures | | 1 | 1 | 1 | 1 | 1 | | 1 | 1 | 1 | 1 | 1 | | 1 | 1 | 1 | 1 | 1 | | 1 | 1 | 1 | 1 | 1 | | 1 | 1 | 1 | 1 | 1 | | 1 | 1 | 1 |
| 1. Subjective outcome | | 4 | 1 | 1 | 4 | 4 | | 4 | 4 | 4 | 3 | 3 | | 3 | 3 | 1 | 3 | 3 | | 3 | 2 | 4 | 1 | 4 | | 1 | 1 | 1 | 3 | 3 | | 1 | 4 | 4 |
| 1. Statistical tests | | 3 | 1 | 1 | 4 | 1 | | 1 | 3 | 1 | 4 | 4 | | 1 | 1 | 1 | 1 | 1 | | 3 | 1 | 2 | 1 | 1 | | 1 | 1 | 1 | 1 | 1 | | 1 | 1 | 1 |
| 1. Confidence intervals | | 4 | 1 | 4 | 4 | 2 | | 4 | 4 | 1 | 4 | 4 | | 4 | 4 | 4 | 1 | 3 | | 4 | 3 | 4 | 4 | 4 | | 4 | 1 | 4 | 4 | 4 | | 4 | 4 | 4 |
| 1. Introduction of bias | | 2 | 1 | 2 | 1 | 1 | | 1 | 2 | 1 | 2 | 2 | | 2 | 1 | 1 | 1 | 1 | | 2 | 2 | 1 | 1 | 1 | | 1 | 1 | 1 | 2 | 1 | | 1 | 1 | 2 |
| **Domain 4: Reference standard** | | | | | | | | | | | | | | | | | | | | | | | | | | | | | | | | | | |
|  | | Acker 1989 | Axelrod 2025 | Birnie 2000 | Currie 1986 | Dalton 1986 | | Dawson 1988 | Feijen 1988 | Gan 2023 | Gonen 1990 | Gough 1986 | | Green 1992 | Hamm 2023 | Horio 1988 | Kerner 2004 | Kitagawa 2000 | | Moore 1990 | Naef 1994 | Nakagawa 2020 | Olesiak-Andryszczak 2025 | Pan 2024 | | Porter 2021 | Porter 2022 | Romano 2009 | Salomon 2004 | Suemitsu 2023 | | Tamaru 2022 | Uzan 1989 | Zizzo 2022 |
| 1. Reference standard | | 3 | 3 | 4 | 4 | 4 | | 3 | 3 | 1 | 3 | 3 | | 3 | 3 | 3 | 3 | 3 | | 3 | 3 | 3 | 3 | 1 | | 1 | 1 | 1 | 3 | 3 | | 3 | 3 | 3 |
| 1. Reference standard appropriate | | 4 | 4 | 4 | 4 | 4 | | 4 | 4 | 2 | 4 | 4 | | 4 | 4 | 4 | 4 | 4 | | 4 | 4 | 4 | 4 | 1 | | 1 | 1 | 3 | 4 | 4 | | 4 | 4 | 4 |
| 1. Blind interpretation | | 4 | 4 | 4 | 4 | 4 | | 4 | 4 | 2 | 4 | 4 | | 4 | 4 | 4 | 4 | 4 | | 4 | 4 | 4 | 4 | 3 | | 3 | 3 | 3 | 4 | 4 | | 4 | 4 | 4 |
| 1. Introduction of bias | | 4 | 4 | 4 | 4 | 4 | | 4 | 1 | 2 | 4 | 4 | | 4 | 1 | 4 | 4 | 4 | | 4 | 4 | 4 | 4 | 2 | | 1 | 2 | 3 | 4 | 4 | | 4 | 4 | 4 |
| **Domain 5: Flow and timing** | | | | | | | | | | | | | | | | | | | | | | | | | | | | | | | | | | |
|  | | Acker 1989 | Axelrod 2025 | Birnie 2000 | Currie 1986 | Dalton 1986 | | Dawson 1988 | Feijen 1988 | Gan 2023 | Gonen 1990 | Gough 1986 | | Green 1992 | Hamm 2023 | Horio 1988 | Kerner 2004 | Kitagawa 2000 | | Moore 1990 | Naef 1994 | Nakagawa 2020 | Olesiak-Andryszczak 2025 | Pan 2024 | | Porter 2021 | Porter 2022 | Romano 2009 | Salomon 2004 | Suemitsu 2023 | | Tamaru 2022 | Uzan 1989 | Zizzo 2022 |
| 1. Appropriate interval | | 4 | 4 | 4 | 4 | 4 | | 4 | 4 | 4 | 4 | 4 | | 4 | 4 | 4 | 4 | 4 | | 4 | 4 | 4 | 4 | 1 | | 1 | 1 | 4 | 4 | 4 | | 4 | 4 | 4 |
| 1. All receive a reference standard | | 4 | 4 | 4 | 4 | 4 | | 4 | 4 | 3 | 4 | 4 | | 4 | 4 | 4 | 4 | 4 | | 4 | 4 | 4 | 4 | 3 | | 3 | 3 | 4 | 4 | 4 | | 4 | 4 | 4 |
| 1. Same reference standard | | 4 | 4 | 4 | 4 | 4 | | 4 | 4 | 4 | 4 | 4 | | 4 | 4 | 4 | 4 | 4 | | 4 | 4 | 4 | 4 | 1 | | 1 | 3 | 4 | 4 | 4 | | 4 | 4 | 4 |
| 1. Complete inclusion | | 2 | 1 | 1 | 2 | 2 | | 1 | 2 | 1 | 4 | 2 | | 2 | 1 | 2 | 1 | 1 | | 1 | 1 | 1 | 1 | 1 | | 1 | 1 | 1 | 1 | 1 | | 1 | 1 | 1 |
| 1. Introduction of bias | | 2 | 2 | 1 | 2 | 2 | | 1 | 2 | 1 | 1 | 2 | | 2 | 1 | 2 | 1 | 1 | | 1 | 1 | 1 | 2 | 1 | | 1 | 2 | 1 | 1 | 1 | | 1 | 1 | 1 |
| **Domain 6: Telemedicine/ Feasibility specific concerns** | | | | | | | | | | | | | | | | | | | | | | | | | | | | | | | | | | |
|  | | Acker 1989 | Axelrod 2025 | Birnie 2000 | Currie 1986 | Dalton 1986 | | Dawson 1988 | Feijen 1988 | Gan 2023 | Gonen 1990 | Gough 1986 | | Green 1992 | Hamm 2023 | Horio 1988 | Kerner 2004 | Kitagawa 2000 | | Moore 1990 | Naef 1994 | Nakagawa 2020 | Olesiak-Andryszczak 2025 | Pan 2024 | | Porter 2021 | Porter 2022 | Kerner 2004 | Romano 2009 | Suemitsu 2023 | | Tamaru 2022 | Uzan 1989 | Zizzo 2022 |
| 1. Receiving or sending environments typical | | 1 | 1 | 1 | 1 | 1 | | 1 | 1 | 1 | 1 | 1 | | 1 | 1 | 1 | 1 | 1 | | 1 | 1 | 1 | 1 | 1 | | 1 | 1 | 1 | 1 | 1 | | 1 | 1 | 1 |
| 1. Level of cost | | 3 | 3 | 4 | 1 | 1 | | 2 | 3 | 3 | 1 | 3 | | 1 | 3 | 3 | 3 | 3 | | 1 | 1 | 3 | 3 | 3 | | 3 | 1 | 3 | 3 | 3 | | 3 | 3 | 1 |
| 1. Technical barriers | | 3 | 1 | 1 | 1 | 1 | | 1 | 1 | 3 | 1 | 1 | | 1 | 1 | 1 | 1 | 3 | | 1 | 3 | 3 | 2 | 2 | | 2 | 2 | 1 | 1 | 3 | | 3 | 1 | 2 |
| **Domain 7: Concerns regarding applicability** | | | | | | | | | | | | | | | | | | | | | | | | | | | | | | | | | | |
|  | | Acker 1989 | Axelrod 2025 | Birnie 2000 | Currie 1986 | Dalton 1986 | | Dawson 1988 | Feijen 1988 | Gan 2023 | Gonen 1990 | Gough 1986 | | Green 1992 | Hamm 2023 | Horio 1988 | Kerner 2004 | Kitagawa 2000 | | Moore 1990 | Naef 1994 | Nakagawa 2020 | Olesiak-Andryszczak 2025 | Pan 2024 | | Porter 2021 | Porter 2022 | Romano 2009 | Salomon 2004 | Suemitsu 2023 | | Tamaru 2022 | Uzan 1989 | Zizzo 2022 |
| 1. Reference standard applicable | | 4 | 4 | 4 | 4 | 4 | | 4 | 4 | 1 | 4 | 4 | | 4 | 4 | 4 | 4 | 4 | | 4 | 4 | 4 | 4 | 1 | | 1 | 1 | 4 | 4 | 4 | | 4 | 4 | 4 |
| 1. Included patients applicable | | 2 | 1 | 1 | 1 | 1 | | 1 | 1 | 1 | 1 | 2 | | 2 | 1 | 1 | 1 | 1 | | 2 | 1 | 1 | 1 | 1 | | 1 | 1 | 1 | 2 | 1 | | 1 | 1 | 1 |
| 1. Conduct of cardiotocography applicable | | 1 | 1 | 1 | 1 | 2 | | 1 | 1 | 1 | 1 | 1 | | 1 | 1 | 1 | 1 | 1 | | 1 | 1 | 1 | 1 | 1 | | 1 | 1 | 1 | 1 | 1 | | 1 | 1 | 1 |
|  | | | | | | | | | | | | | | | | | | | | | | | | | | | | | | | | | | |
| **Qualitative studies** | | | | | | | | | | | | | | | | | | | | | | | | | | | | | | | | | | |
|  | | Aasbo 2024 | Bendix 2024 | Hamm 2024 | Van Den Heuvel 2020 |  | | | | | | | | | | | | | | | | | | | | | | | | | | | | |
| 1. Philosophical congruity | | 1 | 1 | 1 | 1 |  |  |  |  |  |  |  |  |  |  |  |  |  |  |  |  |  |  |  |  |  |  |  |  |  |  |  |  |  |
| 1. Methodology and research question congruity | | 1 | 1 | 1 | 1 |  |  |  |  |  |  |  |  |  |  |  |  |  |  |  |  |  |  |  |  |  |  |  |  |  |  |  |  |  |
| 1. Methodology and data collection congruity | | 1 | 1 | 1 | 1 |  |  |  |  |  |  |  |  |  |  |  |  |  |  |  |  |  |  |  |  |  |  |  |  |  |  |  |  |  |
| 1. Methodology and data analysis congruity | | 1 | 1 | 1 | 1 |  |  |  |  |  |  |  |  |  |  |  |  |  |  |  |  |  |  |  |  |  |  |  |  |  |  |  |  |  |
| 1. Methodology and result interpretation congruity | | 1 | 1 | 1 | 1 |  |  |  |  |  |  |  |  |  |  |  |  |  |  |  |  |  |  |  |  |  |  |  |  |  |  |  |  |  |
| 1. Research culture statement | | 2 | 3 | 3 | 2 |  |  |  |  |  |  |  |  |  |  |  |  |  |  |  |  |  |  |  |  |  |  |  |  |  |  |  |  |  |
| 1. Research influence | | 1 | 1 | 2 | 1 |  |  |  |  |  |  |  |  |  |  |  |  |  |  |  |  |  |  |  |  |  |  |  |  |  |  |  |  |  |
| 1. Participant voices heard | | 1 | 2 | 1 | 1 |  |  |  |  |  |  |  |  |  |  |  |  |  |  |  |  |  |  |  |  |  |  |  |  |  |  |  |  |  |
| 1. Research ethics | | 1 | 1 | 1 | 1 |  |  |  |  |  |  |  |  |  |  |  |  |  |  |  |  |  |  |  |  |  |  |  |  |  |  |  |  |  |
| 1. Flow of research | | 1 | 1 | 1 | 1 |  |  |  |  |  |  |  |  |  |  |  |  |  |  |  |  |  |  |  |  |  |  |  |  |  |  |  |  |  |
|  | | | | | | | | | | | | | | | | | | | | | | | | | | | | | | | | | | |
| **Randomised controlled studies** | | | | | | | | | | | | | | | | | | | | | | | | | | | | | | | | | | |
|  | Domain 1: Randomisation process | | | | | | Domain 2: Deviations from the intended interventions | | | | | | Domain 3: Missing outcome data | | | | | | Domain 4: Measurement of the outcome | | | | | | Domain 5: Selection of the reported result | | | | | | Overall | | | |
| Bekker 2023 | Some concerns | | | | | | Some concerns | | | | | | Low | | | | | | High | | | | | | Low | | | | | | Some concerns | | | |
| Birnie 1997 | Low | | | | | | High | | | | | | Low | | | | | | Low | | | | | | Some concerns | | | | | | High | | | |
| Dawson 1999 | Low | | | | | | Some concerns | | | | | | Low | | | | | | Low | | | | | | Some concerns | | | | | | Some concerns | | | |
| Dawson 1989 | Some concerns | | | | | | High | | | | | | Low | | | | | | Low | | | | | | Some concerns | | | | | | High | | | |
| Monincx 1997 | Some concerns | | | | | | Low | | | | | | Low | | | | | | Low | | | | | | Some concerns | | | | | | Some concerns | | | |
| Wang 2019 | Some concerns | | | | | | High | | | | | | Low | | | | | | Low | | | | | | Some concerns | | | | | | High | | | |
| Zhou 2023 | Some concerns | | | | | | High | | | | | | Some concerns | | | | | | Some concerns | | | | | | Some concerns | | | | | | High | | | |

1 = low risk/yes, 2= unclear risk, 3 = high risk/no, 4 = not applicable.
