# Supplementary material for: Remote home cardiotocography: A systematic review and meta-analysis
Source: PLOS Digit Health. 2026 Jan 12;5(1):e0001184. doi: 10.1371/journal.pdig.0001184 (PMC12795381; doi:10.1371/journal.pdig.0001184)
Supplement: Attachment — Submitted filename: Review_20251024.pdf [file pdig.0001184.s007.pdf]

Review
Manuscript Number PDIG-D-25-00790
"Remote cardiotocography: a systematic review and meta-analysis"

The authors are to be congratulated for a throrough sufficiently detailed Meta-Analysis with clearly presented Figures and Tables on a clinically important topic in antinatal care using adequate statistical analyses. The results support the conclusion of non-inferiority of remote cardiotocography to standard antenatal care. The manuscript should be accepted with wth minor revisions.

Minor issues.

Discussion:
The authors may consider to mention available information on risk stratification and its effect on their conclusion of ‚non-inferiority' of remote cardiotocography,e.g., the use of pregnancy risk scores to define high risk pregnancies that would likely profit from remote CTG monitoring as compared to standard of antinatal care (https://www.sciencedirect.com/science/article/abs/pii/S0301211524005074).

It is likely, that remote CTG monitoring would be superior to the standard of care in the high risk group of pregnancies if tools to predict poor outcome would be employed on a large scale.

Line 604: omit ‚a'
