# Supplementary material for: Remote home cardiotocography: A systematic review and meta-analysis
Source: PLOS Digit Health. 2026 Jan 12;5(1):e0001184. doi: 10.1371/journal.pdig.0001184 (PMC12795381; doi:10.1371/journal.pdig.0001184)
Supplement: Attachment — Submitted filename: Response to reviewers.docx [file pdig.0001184.s008.docx]

Department of Applied Health Sciences

University of Birmingham

17^TH^ November 2025

RE: Manuscript PDIG-D-25-00790

**Dear Dr Leo Anthomy Celi**

Thank you very much for your invaluable input and for considering our article for publication in PLOS Medicine. Please find the reviewer feedback below, with our responses following.

**Reviewer 1**

The topic is highly relevant given the increasing demand for fetal monitoring in high-risk pregnancies and the growing integration of digital health technologies in obstetric practice.

Strengths:
1. The review is comprehensive, covering a wide range of outcomes including clinical, feasibility, diagnostic accuracy, qualitative, and economic aspects.
2. The inclusion of both RCTs and observational studies provides a holistic view of the current evidence.
3. The meta-analysis was appropriately conducted, and the narrative synthesis of heterogeneous observational studies is clear and informative.
4. The authors appropriately highlight the need for standardized outcome reporting and improved methodological rigor in future studies.

- Thank you for your kind comments.

Major Concerns:

1. Low Quality of Evidence:
The GRADE assessment indicating low or very low certainty for all outcomes is a significant limitation. The high risk of bias in many included studies and methodological heterogeneity undermine the strength of the conclusions.

- Thank you for your comment, the authors agree with the limitations of the current body of available research, as evidenced by the GRADE assessments. Limitations in the current research is provided in the discussion, but the authors have added a summary of the GRADE assessments into the discussion on line 603 of the manuscript with tracked changes (line 597 of the clean manuscript).

2. Safety Reporting:
As a clinician, I find the lack of detailed safety protocols and management pathways for abnormal remote CTG traces concerning. Only a minority of studies described follow-up actions, which is critical for clinical implementation.

- Thank you for your comment. The authors agree with this comment, which was why we deemed it was vital to assess the implementation of safety protocols for usage of this type of device within the home setting. This is situated on lines 461-483 of the clean manuscript (lines 467-489 of the manuscript with tracked changes). This is a limitation of the current evidence and why we advocate for further research with rigorous methodology and safety pathways to be conducted. Lack of pathways was written in lines 587-590 in the clean manuscript (593-599 of the manuscript with tracked changes).

3. Generalizability:
All included studies are from high-income countries, limiting the applicability of findings to low-resource settings where remote monitoring could have substantial impact.

- Thank you for your comment. The authors agree with this comment and this limitation is written on lines 663-673 and lines 727-729 of the clean manuscript (lines 669-679 and 733-735 of the tracked changes manuscript).

4. Service-Related Outcomes:
Key outcomes such as emergency department visits, unscheduled hospital admissions, and impact on clinical workflow are underreported. These are essential for evaluating the real-world burden and benefit of remote CTG.

- Thank you for your comment. The authors agree with this comment and information has been written on lines 590-594 (lines 596-599 of the tracked changes manuscript) and lines 639-645 of the clean manuscript (lines 645-651 of the tracked changes manuscript) .

Minor Points:

1. The definition of "interpretable" CTG traces varied widely across studies, which may affect the feasibility conclusions.

- Thank you for your comment. We have included different definitions of an interpretable CTG, provided by the included studies, on lines 376-393 of the manuscript with tracked changes (lines 372-389 of the clean manuscript). Further information has been added into the discussion on lines 576-578 of the manuscript with tracked changes (lines 570-572 of the clean manuscript).

2. Subgroup analyses were planned but not performed due to limited studies, which is understandable but limits the exploration of heterogeneity.

- Thank you for your comment. The authors also recognize this and hope with further future research; this will become feasible to perform. Lack of subgroup analysis is written on lines 357-359 and line 723 of the discussion of the manuscript with tracked changes (lines 353-355 and 717 of the clean manuscript).

Recommendations:

4. Emphasize the need for future RCTs with robust design, clear safety protocols, and standardized outcome sets (e.g., a core outcome set for remote fetal monitoring).

- Thank you for your comment. The authors also recognize this and are currently undertaking an additional systematic review looking at clinical outcome remote for remote obstetric monitoring. A lack of standardized outcome reporting is highlighted in the discussion on lines 628-631 of the manuscript with tracked changes (lines 623-625 of the clean manuscript) and in the conclusion.

5. Encourage studies from low- and middle-income countries to assess feasibility and impact in diverse settings.

- Thank you for your comment. The authors agree with this comment, which is noted in the discussion on lines 669-679 of the manuscript with tracked changes (lines 663-673 of the clean manuscript).

6. Include a clearer discussion on how remote CTG could be integrated into existing antenatal care pathways, including training requirements and clinician acceptance.

- Thank you for your comment. Further information has been added on lines 640-667 of the manuscript with tracked changes (lines 636-661 of the clean manuscript)

Conclusion:

This review provides a valuable synthesis of the current evidence on remote CTG and highlights its potential as a non-inferior alternative to conventional care. However, the low quality of evidence and limited safety reporting preclude strong clinical recommendations at this time. With the suggested revisions, this manuscript will be a significant contribution to the field.

- Thank you very much for your comments, they are greatly appreciated. We hope that what was originally written in the manuscript and predominantly in the discussion, highlighted the main limitations of the current available evidence.

**Reviewer 2**

Abstract

1. Suggest that the statement that “GRADE assessments were low/very low” is expanded to state “GRADE assessments were low/very low quality of evidence”.

- Thank you for your comment, this has been changed on line 37-38 of the clean and tracked changes manuscripts.

Author summary

1. I would suggest rewording the first sentence as it currently sounds as though women self-classify as high-risk and I would specify that this relates to antenatal fetal heart rate monitoring as opposed to intrapartum fetal heart rate monitoring – I think that this placed a demand on maternity rather than obstetric services, as at least in the UK context the majority of these monitorings occur in antenatal day units which are midwifery-led. e.g. “In an era where the number of pregnant women classified as high-risk is increasing, the demand for regular antenatal fetal monitoring has subsequently surged which has increased the number of consultations in outpatient maternity services.”

- Thank you for your comment. The wording has been changed as advised, which can be seen on lines 52-53 in the manuscript with tracked changes.

Introduction

1. Suggest altering the first sentence to “Cardiotocography (CTG) is a commonly used investigative modality in maternity care to evaluate fetal condition; it is frequently used in pregnant women classified as at high-risk.” Is it possible to say at high risk of what? Presumably perinatal mortality?

- Thank you for your comment. The wording has been changed on lines 68-70 in the manuscript with tracked changes.

Re reference

1. Does the office of national statistics data cited include information on high or low-risk status? It certainly doesn’t include the possible reasons outlined in the sentence. It would be good to support this statement with evidence, or make it more clear that this is the authors opinion.

- Thank you for your comment. The national statistics reference was in relation to evidence of an ageing maternal population over recent years. The authors have expanded the sentence slightly (line 74 of the manuscript with tracked changes) and included references of a guideline which have recently changed and lowered their diagnostic thresholds for classifying high risk women. We have also cited an implementation of a national initiative based in the UK. Additionally, the authors have included evidence demonstrating a greater prevalence of pre-existing medical co-morbidities within the pregnant population. References have been re-updated.

2. In line 74, do the authors think it would be reasonable to add the environmental impact of frequent attendance to hospital? The NHS has a plan to reach net zero by 2040, so this is arguably also a relevant impact of frequent episodes of antenatal fetal monitoring.

- Thank you for your comment. We have references the NHS net zero plan on lines 78-79 of the manuscript with tracked changes (line 77-78 of the clean manuscript). References have been updated accordingly.

3. I think it would be good to include a justification why the authors have focussed on the non-inferiority of home CTG to conventional care with respect to clinical and service-led outcomes. Could they make their argument clearer? e.g. “For home CTG monitoring to be an acceptable alternative for service users and clinicians it must have equivalent or non-inferior performance in the identification of fetal compromise and superior participant experience and/or cost-performance.”

- Thank you for your comment. As guided by your comment, we have updated this section of the introduction to include further information between lines 97 – 110 in the manuscript with tracked changes (lines 96-108 of the clean manuscript).

Methods

1. Line 126 – Should this state the Cochrane Database of Clinical Trials? Medline and PubMed search the same database, but the authors did not search Google Scholar or CINAHL. I think the nursing and midwifery database may have had more information about patient experience. I wonder whether it would be worth including these two other sources of evidence.

- Thank you for your comment. We have altered it to the Cochrane Database of Clinical Trials on line 134 of the manuscript with tracked changes (line 132 of the clean manuscript), as it should have correctly been this.
- The authors believe that the searches carried out across the five databases would have achieved enough of a comprehensive search of the available published literature for remote home CTG. As such, the authors have taken a pragmatic approach not to search any more additional databases.

2. Lines 163-165 – I think it would be helpful to understand what the authors assessed in terms of feasibility and clinical utility. Does this mean the proportion of home CTG traces which were recorded or the frequency with which a woman had to attend the maternity unit for a CTG? Or did the authors accept whatever the definition for these outcomes was used in the primary study? I think it would be helpful to add some more detail here.

- Thank you for your comment. Outcomes assessed which related to feasibility and clinical utility have been written on lines 172-176 in the manuscript with tracked changes (lines 170-174 of the clean manuscript).

3. Line 178 – data is a plural, therefore it should read “Continuous outcome data were compared…”

- Thank you for your comment. We have changed this from ‘was’ to ‘were’ on line 189 in the manuscript with tracked changes.

4. Line 202 – the authors state the “database and grey literature searches…” but they do not describe any grey literature searches in the methods section.

- Thank you for your comment. In the methods section on line 136 of the clean manuscript, it reads ‘Reference lists were additionally screened to identify additional grey literature citations eligible for inclusion.’
- Clinicaltrials.gov registry searches is encompassed in the advanced database search on the Cochrane Database of Clinical Trials.

5. Line 227 – should this read “11 studies not disclosing the gestation of monitoring.

- Thank you for your comment, we have updated this as requested on line 237 of the manuscript with tracked changes.

6. Line 300 – I don’t understand what the authors mean by “Results were moderately attributed by a single study”. Do they mean “Results were moderately affected by a single study…” Can the authors clarify this sentence?

- Thank you for your comment, we have changed it to ‘moderately affected’ to improve clarity on line 311 of the manuscript with tracked changes.

7. In multiple places in the results section the authors state “there is a reduction, albeit not statistically significant…” This could reflect their bias or original expectation regarding the results. I would suggest being more careful with language and simply state that there was no statistically significant difference between the two groups.

- Thank you for your comment. We have changed this in the manuscript as advised.

8. I also wonder about the validity of the I-squared statistic with such a small number of studies. [https://bmcmedresmethodol.biomedcentral.com/articles/10.1186/s12874-015-0024-z](https://urldefense.com/v3/__https:/bmcmedresmethodol.biomedcentral.com/articles/10.1186/s12874-015-0024-z__;!!CF15FET90Tp8!DLWYPzwONu7MHYXu_C67nyMRf1THromkZm-Jwx1zMwTQ-SBj0ZRWHXOoz2wxi11PRYgRsN8q_BJRESYI9A$) I wonder whether the authors should either state that there were too few studies to meaningfully calculate heterogeneity (my recommendation) or recognise this in their limitations.

- Thank you for your comment. We have added this on line 279, lines 294-295, and lines 337-338 throughout the results section. Of the manuscript with tracked changes (lines 276-277, lines 291-292 and lines 333-334 of the clean manuscript).

9. Line 388 – What is the total SUS score possible, can the authors report this as 76.5-88.1/Total possible as they have with TUQ score?

- Thank you for your comment. We have added the total SUS score on line 397 of the manuscript with tracked changes. (lines 393 of the clean manuscript).

10. Lines 403-405 “In an era where the number of high-risk obstetric patients continues to rise, remote CTG monitoring may provide an avenue to alleviate the capacity strain on outpatient/inpatient services.” This is interpretation which belongs in the discussion section rather than the results section.

- Thank you for your comment. This sentence has been removed from the results section.

11. Line 455 I think visitation should be visits or attendances.

- Thank you for your comment, this has been changed to attendances on line 462 of the manuscript with tracked changes.

12. In the section on economic burden can the costs be presented in a single currency for comparison?

- Thank you for your comment. The costs have been changed to pound sterling between lines 517 and 534 in the manuscript with tracked changes (lines 514-525 of the clean manuscript).

Discussion

1. Lines 583-585 I think represent the biggest challenge in this field. The diagnostic accuracy was comparable to conventional in hospital CTG. However, there is no evidence that conventional CTG improves perinatal outcome by reducing perinatal mortality. Surely, one of the key points here is that home CTG actually needs to provide some evidence of improved outcome before it can be introduced. Simply performing an ineffective test in another environment doesn’t mean that the technology should be adopted. I think the authors need to address this need to demonstrate improved outcome for women having antenatal CTG. I agree that there is a need for high-quality studies.

- Thank you for your comment. The authors agree that there is minimal evidence to support that conventional CTG reduces perinatal mortality. Whilst perinatal mortality would be objectively difficult to currently assess with home CTG , the authors suggest in the discussion that if home CTG displayed improved healthcare usage outcomes (hospital attendance and clinical workflow), improved environmental impact (as you mentioned below), reduced economic burden and improved clinician/participant acceptability, then this may demonstrate superiority over conventional CTG as a potential care pathway. This is written on lines 97-110 and 648-651 of the manuscript with tracked changes (lines 96-108 and 642-645 of the clean manuscript).

2. I think it is also worth considering the environmental impact of remote monitoring in the discussion section of the manuscript.

- Thank you for your comment. We have added information regarding this on lines 693-703 in the manuscript with tracked changes (lines 687-697 of the clean manuscript).

**Reviewer 3**

The authors are to be congratulated for a thorough sufficiently detailed Meta-Analysis with clearly presented Figures and Tables on a clinically important topic in antenatal care using adequate statistical analyses. The results support the conclusion of non-inferiority of remote cardiotocography to standard antenatal care. The manuscript should be accepted with with minor revisions.

- Thank you very much for your comment.

Minor issues.

Discussion:

The authors may consider to mention available information on risk stratification and its effect on their conclusion of ‚non-inferiority‘ of remote cardiotocography, e.g., the use of pregnancy risk scores to define high risk pregnancies that would likely profit from remote CTG monitoring as compared to standard of antenatal care (https://www.sciencedirect.com/science/article/abs/pii/S0301211524005074). It is likely, that remote CTG monitoring would be superior to the standard of care in the high risk group of pregnancies if tools to predict poor outcome would be employed on a large scale.

- Thank you for your comment. Risk stratification using predictive tools has been added to lines 659-664 in the manuscript with tracked changes (lines 653-658 of the clean manuscript).

Line 604: omit ‚a‘

- Thank you for highlighting this – this has been removed.

**Reviewer 4**

This systematic review comprehensively examined the available literature on home CTG monitoring, including clinical effectiveness, feasibility, diagnostic accuracy, clinical utility, and economic burden. The methods used for the review are appropriate and robust, the language is clear, and the conclusions are supported by the data.

- Thank you for your comment.

Minor comments:

1. In the final sentences of the introduction's second paragraph, the authors differentiate their work from an existing review by emphasizing their focus on home CTG monitoring. To ensure consistency and clarity, could the authors consider aligning the title more directly with this focus, for instance, by using "Home CTG" or "Remote Home CTG" instead of the broader term "Remote CTG"?

- Thank you for your comment. We have changed the title to include to ‘Remote home cardiotocography’.

2. Regarding the grey literature search, the authors mention screening reference lists. Could they please clarify if additional, systematic methods were employed to identify grey literature? Specifically, was any search performed in dedicated grey literature databases such as OpenGrey or clinical trial registries like ClinicalTrials.gov?

- Thank you for your comment. ClinicalTrials.gov was also searched as part of the advanced search using the Cochrane Database of Clinical Trials (CENTRAL). We have updated this in the manuscript to highlight that CENTRAL was a database searched, rather than just writing Cochrane (line 132 of the clean manuscript).

3. In the results section, subsection Randomised controlled trials and GRADE assessment, the authors stated "Despite all assessing remote monitoring in high-risk participants”, however, in Table S5, Wang 2019 (54), High/Low risk patients: NR, please clarify which one is correct?

- Thank you for your comment. We have updated line 274 in the manuscript with tracked changes to say ‘despite all but one study…’.
